# Variational Bayesian inference for logistic regression models for group testing data: Comparing a new hybrid variational inference approach to traditional methods

**DOI:** 10.1007/s11222-026-11013-5

**Published:** 2026-11-05

**Authors:** Xingpei Zhao, Alexander C. McLain, Bo Cai, Dewei Wang, Stella Self

**Affiliations:** 1 Department of Epidemiology and Biostatistics, University of South Carolina, 915 Greene St, Columbia, SC 29208 USA; 2 Department of Statistics, University of South Carolina, 1523 Greene Street, Columbia, SC 29208 USA

**Keywords:** Variational Bayes, Markov chain Monte Carlo, Pooled testing, Binary outcomes

## Abstract

Group testing is an efficient means of screening for infectious disease that combines specimens from multiple individuals and tests them as a single ‘pooled’ specimen. If a pool tests positive, subsequent tests are usually conducted to determine the disease status of the contributing individuals; if a pool tests negative, all samples in the pool are considered disease free. In recent years, Bayesian regression frameworks for group testing data have been developed, offering flexibility across group testing protocols and enabling the estimation of unknown assay accuracies. However, existing Bayesian methods rely heavily on Markov chain Monte Carlo (MCMC) sampling for estimation and inference, which becomes increasingly computationally inefficient as sample sizes grow, creating a bottleneck that limits the applicability of these models to large datasets. Variational Bayes (VB), an optimization-based algorithm that provides an analytical approximation to the posterior distribution, offers a more computationally efficient alternative to MCMC. To overcome the computational challenges of current Bayesian group testing methods, we develop a VB algorithm for group testing regression. Because VB methods are known to underestimate the posterior variance, we also propose an alternative inference method that exploits the asymptotic normality of VB estimators. We demonstrate with numerical studies that this hybrid inference approach achieves comparable inference to the MCMC approach while being more computationally scalable. We then apply the proposed VB method to COVID-19 group testing data from 43,185 individuals in the National Institutes of Health’s All of Us Research Program.

**Supplementary Information:**

The online version contains supplementary material available at https://doi.org/10.1007/s11222-026-11013-5.

## Introduction

Group testing is a disease screening procedure that combines specimens obtained from multiple individuals and tests them as pooled specimens (Dorfman 1943). If the test result for a pooled specimen is negative, all individuals within that specific pool are usually deemed free of the disease or infection under consideration. Conversely, if the test yields a positive result for a pooled specimen, we generally conclude that at least one individual within that group has the disease or is infected. Individuals contributing to positive pools are often retested to determine their disease status. Under relatively low disease prevalence, group testing can substantially reduce the number of diagnostic tests required and improve overall testing efficiency. During the COVID-19 pandemic, the high demand for testing and limited supply of testing materials brought renewed attention to group testing procedures. For example, several researchers evaluated group testing protocols for COVID-19 surveillance in both clinical practice (Schmidt et al. 2020; Ben-Ami et al. 2020; Yelin et al. 2020) and numerical experiments (Eberhardt et al. 2020; Deckert et al. 2020; Barak et al. 2021; Comess et al. 2022), confirming that multi-stage group testing protocols can reduce the number of tests needed by 67%–86% and 58%–89% under infection prevalences of 5% and 1%, respectively.

Modeling and drawing statistical inferences from group testing data presents some unique challenges. First, sometimes only pooled responses are observed, though depending on the group testing protocol, individual retesting responses from positive pools may also be available. Second, these testing responses may be prone to error (i.e., subject to false positives or false negatives). Recent work has focused on a class of Bayesian group testing regression models which address these complexities (Mcmahan et al. 2017; Joyner et al. 2020; Liu et al. 2020; Huang et al. 2024; St. Ville, M.E., McMahan, C.S., Bible, J.D., Tebbs, J.M., Bilder, C.R., 2025). These flexible Bayesian approaches are suitable for any group testing protocol and allow for the estimation of unknown assay accuracy parameters. Existing methods for fitting these models rely heavily on Markov chain Monte Carlo (MCMC) sampling to obtain a sample from the posterior distribution. These MCMC algorithms require sampling latent individual disease statuses that follow conditional Bernoulli distributions. These conditional distributions depend on the disease status of other individuals in the same testing pool and therefore require serial updates. Thus, the computation time of the MCMC procedure increases noticeably as the number of individuals increases. This computational bottleneck limits the application of Bayesian regression models to large group testing data sets. This project aims to develop scalable inference for Bayesian group testing regression models.

Variational Bayesian inference, or variational Bayes (VB) is a method inspired by machine learning techniques that has gained popularity as a technique for approximating the posterior distribution of Bayesian models (Jordan et al. 1999; Wainwright and Jordan 2008). VB methods rely on optimization to provide an analytical approximation to the posterior distribution and are generally more computationally efficient than MCMC algorithms. VB algorithms aim to minimize the Kullback–Leibler (KL) divergence (Kullback and Leibler 1951) between a tractable family of distributions and the true posterior distribution. This process is equivalent to maximizing the evidence lower bound (ELBO) on the approximation distribution (Blei et al. 2017).

VB algorithms commonly take the class of tractable distributions to be the mean-field variational family, so that the variational (approximation) distribution can be factorized over groups of parameters that are mutually independent. VB is most efficient for conditional conjugate models, in which both parameters and any necessary latent variables belong to the exponential family and parameters have conjugate priors. Under these conditions, the simple coordinate ascent variational inference (CAVI) algorithm can be implemented (Bishop 2006) to perform optimization. The CAVI algorithm sequentially maximizes the ELBO with respect to each variational factor while holding the remaining factors fixed at their most recent updates; the iterations proceed until the ELBO converges.

Recent work by Durante and Rigon (2019) formally extended the framework of conditionally conjugate mean-field VB to Bayesian logistic regression by integrating the Pólya–gamma (PG) data augmentation scheme for logistic models (Polson et al. 2013) into the seminal variational algorithm originally proposed by Jaakkola and Jordan (2000). Their work clarifies the theoretical connection between PG data augmentation and the Jaakkola–Jordan bound, establishing a formal VB framework for logistic models that accommodates the CAVI algorithm. These insights highlight the potential for extending VB for logistic models to more complex regression settings involving binary data, such as group testing data with binary outcomes. The goal of our work is threefold: to develop variational Bayesian inference for Bayesian group testing models; to develop an alternate VB inference approach that overcomes the shortcomings of traditional VB inference; and to compare the estimation accuracy, inferential reliability and computational cost of the first two methods to full Bayesian inference implemented via MCMC methods.

VB is well-known to underestimate the true posterior variance of model parameters (Blei et al. 2017; Durante and Rigon 2019). This arises from VB’s reliance on the KL divergence as the objective function. To overcome this limitation, we propose an alternate ‘hybrid’ inference approach that exploits the asymptotic normality of VB posterior mean estimates. Assuming that data come from a distribution with true (non-random) parameters, Wang and Blei (2019) extend the classical Bernstein-von Mises theorem (Van Der Vaart 1998) and Bernstein-von Mises theorem under misspecification (Kleijn and van der Vaart 2012) to VB approximation. This variational Bernstein-von Mises theorem demonstrates that under the usual assumptions necessary for the classical Bernstein-von Mises theorem, the VB approximation is consistent and converges in total variation distance to the KL minimizer of a normal distribution with a random mean centered at the true parameter values. Additionally, it also proves that the VB mean estimators are consistent and asymptotically normal.

According to the classical Bernstein-von Mises theorem, the posterior distribution converges in total variation distance to a normal distribution whose covariance matrix is the inverse of the Fisher information matrix evaluated at the true parameter values. The variational Bernstein-von Mises theorem further establishes that the VB estimator is consistent and asymptotically normal. Consequently, we propose to approximate the asymptotic covariance matrix of the VB mean estimator using the inverse Fisher information matrix evaluated at the mean of the optimal VB approximation distribution. We note that this matrix is used to approximate the (frequentist) sampling distribution of the VB mean estimators, rather than to approximate the posterior distribution of the model parameters. We use the diagonal elements of this matrix to estimate the sampling variance of the VB mean estimator and produce Wald confidence intervals in the usual way. We demonstrate with numerical studies that this approach produces Wald confidence intervals that maintain their nominal levels, suggesting that these diagonal elements are a good approximation to the true asymptotic variance of the sampling distribution of the optimal VB mean estimator. We find this hybrid inference approach performs favorably when compared to both traditional VB inference and full MCMC-based inference.

The remainder of this article is outlined as follows. In Sect. 2, we introduce notations, describe the VB algorithm for group testing data, formalize the algorithm in detail, and present our hybrid inference approach. In Sect. 3, we conduct a simulation study to compare the performance of traditional VB inference, our hybrid inference approach and full MCMC-based inference. In Sect. 4, we apply the traditional VB approach and the hybrid approach to the COVID-19 data from National Institutes of Health’s All of US research program. Finally, we draw conclusions and suggest areas for future work in Sect. 5.

## Methods

### Notations and preliminaries

Suppose we have tested *N* individuals for a disease or infection using a group testing protocol. We allow for a very general testing protocol, requiring only that each individual is tested in at least one pool and allowing for (though not requiring) multi-stage testing procedures and the retesting of individuals in positive pools. We assume that we have observed *J* testing responses $\boldsymbol{Z} = (Z_1, \dots , Z_J)^\top$ and allow $\mathcal {P}_j \subset \{1,2,\dots ,N\}$ to denote the set of individuals who contributed to pool *j*. Note that under this notation, any individual retest results are included in $\boldsymbol{Z}$ as ‘pools’ of size 1.

We further assume that we have covariate information on the *N* individuals; allow $\boldsymbol{x}_i = (1, x_{i1}, \dots , x_{ip})^\top$ to be the p+1 dimensional vector of covariates associated with individual *i*. In order to relate this covariate information to the individuals’ infection statuses, we introduce $\tilde{\boldsymbol{Y}}= (\tilde{Y}_1, \dots , \tilde{Y}_N)^\top$ as the true infection statuses for the *N* individuals, where $\tilde{Y}_i=1$ indicates that the individual is infected and $\tilde{Y}_i=0$ indicates non-infected. We note that $\tilde{\boldsymbol{Y}}$ is unobserved due to the pooled nature of the observed data and the fact that the assays used may be subject to errors. Following Mcmahan et al. (2017), we assume that the $\tilde{Y}_i$ independently follow Bernoulli distributions with $P(\tilde{Y}_i = 1) = g^{-1}(\boldsymbol{x}_i^\top \boldsymbol{\beta })$, where g(·) is assumed to be a standard logit link. These latent individual statuses naturally give rise to latent pool statuses, denoted $\boldsymbol{\tilde{Z}} = (\tilde{Z}_1, \dots , \tilde{Z}_J)^\top$, where $\tilde{Z}_j = \mathbb {I}\left( \sum _{i\in \mathcal {P}_j} \tilde{Y}_i > 0 \right)$ equals 1 if pool *j* contains at least one truly infected individual and equals 0 otherwise. We allow for imperfect testing, and the accuracy of testing is measured by *sensitivity* $Se_j = P(Z_j = 1 \mid \tilde{Z}_j=1)$ and *specificity* $Sp_j = P(Z_j = 0 \mid \tilde{Z}_j=0)$. We assume testing has been conducted using *M* different assays, each characterized by a distinct unknown sensitivity and specificity. Define the index set $\mathcal {M}_m$ as the set of indices for pools tested by the *m*th assay. Let $Se_{(m)}$ and $Sp_{(m)}$ denote the sensitivity and specificity of the *m*th assay, respectively, so that $Se_j$ = $Se_{(m)}$ and $Sp_j = Sp_{(m)}$ for all $j \in \mathcal {M}_m$.

As is typical in group testing regression models, we assume observed pool responses $\boldsymbol{Z}$ are mutually independent given the true pool statuses $\boldsymbol{\tilde{Z}}$ and that the distribution of $\boldsymbol{Z} \mid \boldsymbol{\tilde{Z}}$ does not depend on covariates. Under these assumptions and following Mcmahan et al. (2017), we have the following likelihood function:

1 $$\begin{aligned}&p\left( \boldsymbol{Z}, \tilde{\boldsymbol{Y}} \mid \boldsymbol{\beta }, \boldsymbol{Se}, \boldsymbol{Sp} \right) \nonumber \\&\quad = \prod ^{J}_{j=1} \left[ Se_j ^{Z_j} \left( 1-Se_j\right) ^{1-Z_j}\right] ^{\tilde{Z_j}} \nonumber \\&\qquad \; \; \; \times \left[ Sp_j ^{1-Z_j} \left( 1-Sp_j\right) ^{Z_j}\right] ^{1-\tilde{Z_j}} \nonumber \\&\qquad \; \; \; \times \prod ^{N}_{i=1} \left[ g^{-1}\left( \boldsymbol{x}_i^\top \boldsymbol{\beta } \right) \right] ^{\tilde{Y}_i} \left[ 1-g^{-1}\left( \boldsymbol{x}_i^\top \boldsymbol{\beta }\right) \right] ^{1-\tilde{Y}_i}, \end{aligned}$$

where $$\boldsymbol{Se} = (Se_{(1)},...,Se_{(M)})^\top $$ and $$\boldsymbol{Sp} = (Sp_{(1)},...,Sp_{(M)})^\top $$.

To facilitate estimation and inference, we adopt the Bayesian paradigm. We intend to develop a VB approach for estimation and inference using the CAVI algorithm to find the optimal variational distribution. Such an approach requires a conditionally conjugate model, which can be achieved in our case by implementing the Póyla-gamma data augmentation method of Polson et al. (2013). Specifically, we introduce latent random variables $\boldsymbol{\omega }= \left( \omega _1, \dots , \omega _N \right) ^\top$ so that the $\omega _i$s independently follow a PG(0, 1) distribution. Our augmented likelihood function thus becomes:

2 $$\begin{aligned}&p\left( \boldsymbol{Z}, \tilde{\boldsymbol{Y}}, \boldsymbol{\omega } \mid \boldsymbol{\beta }, \boldsymbol{Se}, \boldsymbol{Sp} \right) \nonumber \\&\quad \propto \prod ^{J}_{j=1} \left[ Se_j ^{Z_j} \left( 1-Se_j\right) ^{1-Z_j}\right] ^{\tilde{Z_j}} \nonumber \\&\quad \; \quad \; \; \times \left[ Sp_j ^{1-Z_j} \left( 1-Sp_j\right) ^{Z_j}\right] ^{1-\tilde{Z_j}} \nonumber \\&\quad \; \quad \; \; \times \exp \left[ -\frac{1}{2}(\boldsymbol{h}- X\boldsymbol{\beta })^\top \Omega (\boldsymbol{h}-X\boldsymbol{\beta })\right] \nonumber \\&\quad \prod _{i=1}^N f_{\omega }\left( \omega _i\right) , \end{aligned}$$

where $\boldsymbol{h} = \left( \kappa _1/\omega _1, \dots , \kappa _N/\omega _N \right) ^\top$, $\kappa _i = \tilde{Y}_i - 1/2$, and $\Omega =\text {diag}\left( \omega _1,\dots ,\omega _N \right)$. The matrix $X=(\boldsymbol{x}_1^\top , \dots \boldsymbol{x}_N^\top )$ is an N×(p+1) design matrix. The function $f_{\omega }$ is defined as $f_{\omega }\left( \omega _i\right) =\exp \left( \kappa _i^2 / 2 \omega _i\right) p\left( \omega _i\right)$, where $p(\omega _i)$ denotes the density function of a PG random variable $\omega _i \sim \operatorname {PG}(1,0)$. We then assign the following prior distributions to complete the conditionally conjugate model:

$$\begin{aligned}&\boldsymbol{\beta } \sim \mathcal {N}(\boldsymbol{\mu }_0,\Sigma _0) \\&Se_{(m)} \sim \operatorname {Beta}(a_{e(m)}, b_{e(m)}), \text { for } m=1,\dots , M \\&Sp_{(m)} \sim \operatorname {Beta}(a_{p(m)}, b_{p(m)}), \text { for } m=1,\dots , M . \end{aligned}$$

### Proposed variational representation

Recall that VB algorithms aim to minimize the KL divergence between a class of ‘tractable’ distributions *Q* and the true posterior distribution. The optimal variational distribution $q^*$ is defined by

3 $$\begin{aligned} &  q^*(\tilde{\boldsymbol{Y}}, \boldsymbol{\beta }, \boldsymbol{\omega }, \boldsymbol{Se}, \boldsymbol{Sp}) \nonumber \\ &  \quad = \text {argmin}_{q \in Q} \textrm{KL} \Big [ q (\tilde{\boldsymbol{Y}}, \boldsymbol{\beta }, \boldsymbol{\omega }, \boldsymbol{Se}, \boldsymbol{Sp}) \Vert \nonumber \\ &  \quad p( \tilde{\boldsymbol{Y}}, \boldsymbol{\beta }, \boldsymbol{\omega }, \boldsymbol{Se}, \boldsymbol{Sp} \mid \textbf{Z} ) \Big ]. \end{aligned}$$

Following Durante and Rigon (2019), we assume that *Q* is the mean-field variational family and that the variational distribution of the unknown parameters and latent variables can be factorized as

4 $$\begin{aligned} &  q (\tilde{\boldsymbol{Y}}, \boldsymbol{\beta }, \boldsymbol{\omega }, \boldsymbol{Se}, \boldsymbol{Sp}) \nonumber \\ &  \quad = q(\boldsymbol{\beta }) \prod ^{N}_{i=1}q(\omega _i)q(\tilde{Y}_i)\prod ^{M}_{m=1}q(Se_{(m)}) q(Sp_{(m)}). \end{aligned}$$

The CAVI algorithm finds the optimal variational distribution $q^*$ by successively maximizing the ELBO with respect to each variational factor in $q(\boldsymbol{\beta })$, $q(\tilde{Y}_i)$, $q(\omega _i)$, $q(Se_{(m)})$, and $q(Sp_{(m)})$ while fixing the other factors at their most recent updates.

There is usually a tradeoff between the simplicity of the assumed variational factorization and the accuracy with which the optimal variational distribution can approximate the true posterior distribution. We note that our proposed factorization allows for dependency between the components of $\boldsymbol{\beta }$. It is also possible to allow dependency in the variational distribution of the $\tilde{Y}_i$s for individuals tested in the same pool, though the exact nature of this factorization depends on the group testing protocol employed. We provide details for two common group testing protocols in Appendix A.1. Finally, we note that in the true posterior distribution, the assay accuracy parameters are conditionally independent from one another and from the regression coefficients given $\boldsymbol{\tilde{Y}}$. Therefore, assuming a variational distribution specified in Eq. 4 is functionally equivalent to assuming $\prod _{i=1}^N q(\omega _i) q(\tilde{Y}_i)q(\boldsymbol{\beta },\boldsymbol{Se}, \boldsymbol{Sp})$, as the two factorizations produce the same optimal variational distribution.

The optimal variational factors at each iteration *t* of the CAVI algorithm are provided below. For the regression coefficients $\boldsymbol{\beta }$, we have:

$$\begin{aligned} &  q^{(t)}(\boldsymbol{\beta }) \propto \operatorname {exp}\left\{ \mathbb {E}_{\tilde{\boldsymbol{Y}},\boldsymbol{\omega }, \boldsymbol{Se}, \boldsymbol{Sp}} \operatorname {log}p(\boldsymbol{\beta }|\boldsymbol{\tilde{Y}}, \boldsymbol{\omega }, \boldsymbol{Se}, \boldsymbol{Sp}, \boldsymbol{Z}) \right\} \\\ &  \quad \propto \mathcal {N}_p \left( \boldsymbol{\mu }^*_{b}, \Sigma ^*_b \right) , \end{aligned}$$

where

$$ \begin{aligned} \boldsymbol{\mu }^*_{b}&= \left( X^{\top } \mathbb {E}(\Omega ) X+\Sigma _0^{-1}\right) ^{-1}\left[ \Sigma _0^{-1} \boldsymbol{\mu }_0+X^{\top } \mathbb {E}(\boldsymbol{\kappa })\right] \\ \Sigma ^{*}_b&= \left( X^{\top } \mathbb {E}(\Omega ) X+\Sigma _0^{-1}\right) ^{-1}, \\ \end{aligned} $$

$\mathbb {E}(\Omega ) = \text {diag}\left( \mathbb {E}(\omega _1) \dots , \mathbb {E}(\omega _N) \right)$ and $\mathbb {E}(\boldsymbol{\kappa }) = (\mathbb {E}(\tilde{Y}_1) - 1/2, \dots , \mathbb {E}(\tilde{Y}_N) - 1/2 )^{\top }$. For the latent PG random variables, we have:

$$\begin{aligned} &  q^{(t)}(\omega _i) \propto \operatorname {exp}\left\{ \mathbb {E}_{\boldsymbol{\beta }, \tilde{\boldsymbol{Y}},\boldsymbol{Se}, \boldsymbol{Sp}} \operatorname {log} p(\omega _i | \boldsymbol{\beta }, \boldsymbol{\tilde{Y}}, \boldsymbol{Se}, \boldsymbol{Sp}, \boldsymbol{Z}) \right\} \\ &  \qquad \propto \operatorname {PG} \left( 1, \xi _i \right) , \end{aligned}$$

where $$\xi _i = (\phi _i)^{1/2}$$ and $$\phi _i = \boldsymbol{x}_i^{\top }\Sigma _b^{*} \boldsymbol{x}_i + (\boldsymbol{x}_i^{\top } \boldsymbol{\mu }^{*}_b)^2$$.

For the assay accuracy parameters, we have:

$$ \begin{aligned} q^{(t)}(Se_{(m)})&\propto \exp \left\{ \mathbb {E}_{\boldsymbol{\beta }, \tilde{\boldsymbol{Y}}, \boldsymbol{\omega }, \boldsymbol{Sp}, \boldsymbol{Z}} \log p(Se_m| \boldsymbol{\beta }, \tilde{\boldsymbol{Y}}, \boldsymbol{\omega }, \boldsymbol{Sp}) \right\} \\&\propto \operatorname {Beta}\left( a^{*}_{e(m)}, b^{*}_{e(m)} \right) \\ q^{(t)}(Sp_{(m)})&\propto \exp \left\{ \mathbb {E}_{\boldsymbol{\beta }, \tilde{\boldsymbol{Y}}, \boldsymbol{\omega }, \boldsymbol{Se}} \log p(Sp_m| \boldsymbol{\beta }, \tilde{\boldsymbol{Y}}, \boldsymbol{\omega }, \boldsymbol{Se}, \boldsymbol{Z} \right\} \\&\propto \operatorname {Beta}\left( a^{*}_{p(m)}, b^{*}_{p(m)} \right) , \end{aligned} $$

where

$$ \begin{aligned} a^{*}_{e(m)}&= \sum _{j \in \mathcal {M}_m} Z_j \mathbb E(\tilde{Z}_j)+a_{e(m)} \\ b^{*}_{e(m)}&= \sum _{j \in \mathcal {M}_m}\left( 1-Z_j\right) \mathbb E (\tilde{Z}_j)+b_{e(m)}\\ a^{*}_{p(m)}&= \sum _{j \in \mathcal {M}_m}\left( 1-Z_j\right) \left( 1-\mathbb E(\tilde{Z}_j)\right) +a_{p(m)} \\ b^{*}_{p(m)}&= \sum _{j \in \mathcal {M}_m} Z_j\left( 1-\mathbb E(\tilde{Z}_j)\right) +b_{p(m)}. \end{aligned} $$

The optimal variational factors for the latent individual statuses are Bernoulli distributions, but their exact form depends on the group testing protocol implemented. We note that the dependence structure between the $\tilde{Y}_i$’s in the true posterior depends on the group testing protocol used and can be quite complex. The degree to which the assumed factorization provides a good approximation to the true posterior likely varies from one protocol to another, and it is possible to assume a factorization which allows for dependence between $\tilde{Y}_i$s for individuals tested in the same pool. Appendix A.1 provides details for such a factorization and the associated variational distributions under two common group testing protocols. We also provide expressions for the individual testing case for comparison purposes. Finally, we note that the optimal variational factors for the $\tilde{Z}_j$s (necessary for computing the $\mathbb E(\tilde{Z}_j)$s) may be easily obtained from the $q^{(t)}(\tilde{Y}_i)$s.

The process outlined in Algorithm 1 delineates the steps of the CAVI algorithm. Additional details for computing the necessary expectations are provided in Appendix A.2. A small threshold, denoted ϵ, (e.g. $\epsilon = 10^{-3}$) is used as the stopping criteria for the CAVI algorithm. The algorithm terminates when the change in the ELBO is less than ϵ or when the maximum number of iterations, such as 50,000, is reached. Convergence is assessed based on the stability of the VB estimators, for example, when the changes in any estimators between iterations are less than $10^{-3}$.

**Algorithm 1:**
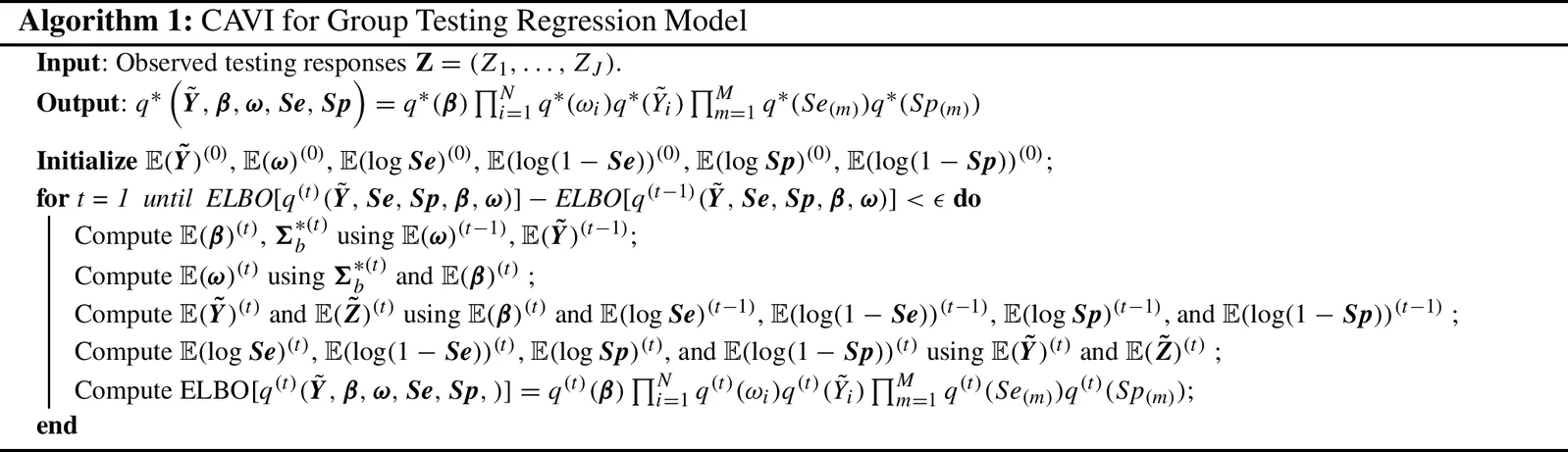
CAVI for Group Testing Regression Model

### Traditional VB inference approach

Traditionally, VB methods use the mean of the optimal variational distribution $q^*$ to estimate the posterior mean of the unknown parameters. The posterior variance is approximated with the variance of $q^*$, though this approach is well-known to underestimate the true posterior variance (Blei et al. 2017; Durante and Rigon 2019). In this work, we perform ‘traditional’ VB inference by constructing 95% equal-tailed credible intervals for the parameters using the 0.025 and 0.975 quantiles of the optimal variational distribution. These intervals are Bayesian credible intervals, though they rely on an approximation to the posterior distribution to quantify the uncertainty in the parameters rather than using the true posterior distribution.

### Hybrid inference approach

Wang and Blei (2019) extend the classical Bernstein-von Mises theorem to VB approximation by assuming data arise from a distribution governed by a set of ‘true’ (non-random) parameters, denoted $\boldsymbol{\theta _0}$. Under this framework, the data generation process is (conceptually) frequentist, though a Bayesian model is fit to the data. The limiting behavior of the optimal VB variational distribution under this framework is then considered. Under some rather technical assumptions (see assumption 1 of (Wang and Blei 2019) for details), the variational Bernstein-von Mises establishes that the optimal VB variational distribution converges in total variation to the KL minimizer of a random normal distribution centered at the true parameter values. This theorem has consequences for the sampling distribution of the *mean* of the optimal variational distribution (denoted $\boldsymbol{\hat{\theta }_{VB}}$) which we consider here. For our purposes, we note that Assumption 1.3 of Wang and Blei (2019) guarantees the local asymptotic normality (LAN) of the variational log-likelihood (see Sections 1.1 and 3.4 of Wang and Blei (2019)). We allow $\Delta _{n, \theta _0}$ and $V_{\theta _0}$ to denote the sequence of random vectors and associated non-singular matrix whose existence satisfies the LAN assumption, and refer to the distribution of $\Delta _n$ as $d_n$. If *Q* is the mean field variational family and $d_n$ converges in distribution to *d*, the sampling distribution of $\boldsymbol{\hat{\theta }_{VB}}$ also converges in distribution to *d*. For ‘well-behaved’ models, *d* is a normal distribution with mean $\boldsymbol{\theta }_0$ and covariance matrix $V_{\theta _0}^{-1}$. In general, deriving the exact form for $V_{\theta _0}^{-1}$ is difficult, but this matrix is often closely related to the inverse Fisher Information matrix. We therefore propose to approximate *d* with $N(\boldsymbol{\theta }_0, \mathcal I(\boldsymbol{\theta _0})^{-1})$, where $\mathcal I(\boldsymbol{\theta })$ is the Fisher information matrix evaluated at $\boldsymbol{\theta }$.

Due to the presence of latent data in our model, we use Louis’s method to obtain the observed Fisher information matrix via

5 $$\begin{aligned} \begin{aligned} \mathcal {I}(\boldsymbol{\theta })&{=} -\mathbb {E} \left\{ \frac{\partial ^2}{\partial \boldsymbol{\theta } \partial \boldsymbol{\theta }^\top } \mathcal {L} (\boldsymbol{\theta }\mid \textbf{Z}) \mid \textbf{Z}\right\} \\&= -\mathbb {E} \left\{ \frac{\partial ^2}{\partial \boldsymbol{\theta } \partial \boldsymbol{\theta }^\top } \mathcal {L} (\boldsymbol{\theta }\mid \tilde{\boldsymbol{Y}}, \textbf{Z}) \mid \textbf{Z} \right\} \\&- \text {cov} \left\{ \frac{\partial }{\partial \boldsymbol{\theta }} \mathcal {L} (\boldsymbol{\theta }\mid \tilde{\boldsymbol{Y}}, \textbf{Z})\mid \textbf{Z} \right\} . \end{aligned} \end{aligned}$$

In the context of the regression models for group testing data with unknown accuracy probabilities, we define the parameter vector $\boldsymbol{\theta } = \left( \boldsymbol{\beta }, \boldsymbol{Se}, \boldsymbol{Sp} \right) ^\top$, though this can be simplified to $\boldsymbol{\theta } = \boldsymbol{\beta }$ if $\boldsymbol{Se}$ and $\boldsymbol{Sp}$ are known. Here the log-likelihood $\mathcal {L}(\boldsymbol{\theta } \mid \tilde{\boldsymbol{Y}}, \textbf{Z})$ may be obtained by taking the log of Eq. 1.

In practice, $\boldsymbol{\theta _0}$ is unknown, so we estimate $\mathcal I(\boldsymbol{\theta _0})^{-1}$ with $\mathcal I(\boldsymbol{\hat{\theta }_{VB}})^{-1}$, where $\mathcal {I}(\boldsymbol{\hat{\theta }_{VB}})$ is obtained by evaluating Eq. 5 at the VB mean estimates $\boldsymbol{\hat{\theta }_{VB}}$ and $\boldsymbol{\tilde{Y}_{VB}}$. Further details on the calculation of this covariance matrix $\Sigma _{q^*}$ can be found in Appendix B. Under our ‘hybrid’ inference approach, we compute a 95% Wald confidence interval using $\boldsymbol{\hat{\theta }_{VB}}$ as our point estimator and the square root of the corresponding diagonal entry of $\Sigma _{q^*}$ as the standard deviation.

The case in which $\boldsymbol{Se}$ and $\boldsymbol{Sp}$ are treated as unknown parameters deserves special attention. In the true posterior distribution, there is dependence among $\boldsymbol{\beta }$, $\boldsymbol{Se}$ and $\boldsymbol{Sp}$ induced by their shared dependence on $\boldsymbol{\tilde{Y}}$. However, the proposed VB factorization assumes $\boldsymbol{\tilde{Y}}$ is independent from $\boldsymbol{\beta }$, $\boldsymbol{Se}$ and $\boldsymbol{Sp}$ and as a consequence, that $\boldsymbol{\beta }$, $\boldsymbol{Se}$ and $\boldsymbol{Sp}$ are independent from one another. The traditional VB inference approach therefore does not capture the dependence in these parameters or the additional variability in these parameters induced by their dependence on $\boldsymbol{\tilde{Y}}$. However, the expression for $\mathcal I(\boldsymbol{\theta })$ is derived from the original likelihood function, not the assumed VB factorization. As a result, $\mathcal I(\boldsymbol{\theta })$ accounts for the covariances between $\boldsymbol{\beta }$, $\boldsymbol{Se}$ and $\boldsymbol{Sp}$ through its diagonal elements (see Appendix B for more details). Therefore, provided that $\boldsymbol{\hat{\theta }_{VB}}$ is a good approximation for $\boldsymbol{\theta _0}$, we expect $\mathcal I( \boldsymbol{\hat{\theta }_{VB}})$ to sufficiently capture both the dependence between $\boldsymbol{\hat{\beta }_{VB}}$, $\boldsymbol{ \hat{S}e_{VB}}$ and $\boldsymbol{\hat{S}p_{VB}}$ and the (additional) variance of $\boldsymbol{ \hat{\beta }_{VB}}$, $\boldsymbol{ \hat{S}e_{VB}}$ and $\boldsymbol{\hat{S}p_{VB}}$ arising from their dependence on the (unknown) $\boldsymbol{\tilde{Y}}$. This hypothesis is supported by the hybrid inference approach out-performing the traditional inference approach in simulation settings (see Sect. 3).

## Simulation study

In this section, we evaluate the performance of the proposed VB method under different group testing protocols. We compare the estimation accuracy of the VB mean estimators to posterior means obtained via a Gibbs-based MCMC algorithm which also exploits the PG data augmentation scheme. We also compare the inferential capabilities of traditional VB inference, our hybrid approach and full MCMC-based inference, as well as comparing the computation time of the three approaches. We consider three different group testing protocols: Dorfman testing (DT), master pool testing (MPT) and individual testing (IT, i.e. traditional testing). Under MPT, each individual is assigned to exactly one pool and no retesting is performed. DT is similar to MPT, except that contributors to positive pools are individually retested. Since the assay accuracy parameters are weakly identifiable without retesting information, $\boldsymbol{Se}$ and $\boldsymbol{Sp}$ are assumed to be known for the MPT and IT protocols but estimated under the DT protocol.

### Simulation settings

For each simulation setting, we generate the true individual disease statuses from a Bernoulli model as follows:

$$\begin{aligned} \operatorname {logit} \left[ \text {Pr}\left( \tilde{Y}_i = 1\right) \right] = \beta _0 + \beta _1 x_{1i} + \beta _2 x_{2i}, \end{aligned}$$

where $\boldsymbol{\beta }= \left( -4,2,1 \right) ^\top$, $x_{1i}$ is generated from a standard normal distribution, $x_{1i} \sim \mathcal {N}(0,1)$ and $x_{2i}$ follows a Bernoulli distribution with the probability of 0.5, $x_{2i} \sim \operatorname {Bernoulli}(0.5)$. The overall prevalence of infection is approximately 10%. We consider five sample sizes $N \in \{ 300, 1500, 3000, 6000, 12000\}$. We assume that we conduct testing using M=2 assays, one for pool testing and the other for individual testing. We set sensitivity ($\boldsymbol{Se}$) for pool and individual testing to 0.95 and 0.98 respectively, with $Se_{(1)} =0.95, Se_{(2)}=0.98$, and specificity ($\boldsymbol{Sp}$) for pool and individual testing to 0.98 and 0.99 respectively with $Sp_{(1)} =0.98, Sp_{(2)}=0.99$. We consider two pools sizes (3 and 10) for the DT and MPT protocols and ‘pools’ of size 1 for the IT protocol. After randomly assigning individuals to pools, we compute $\tilde{Z}_j = I(\sum _{i\in \mathcal P_j} \tilde{Y}_i >0)$ and then generate $Z_j \sim$ Bernoulli$(\tilde{Z}_j Se_{(m)} + (1-\tilde{Z}_j)(1-Sp_{(m)}))$ where m=1 for DT and MPT and m=2 for IT. For the DT scenario, we also simulate retesting as follows. For every *j* with $Z_j = 1$ and every $i \in \mathcal P_j$, we compute $Z_{j_i}\sim$ Bernoulli$(\tilde{Y}_i Se_{(2)} + (1-\tilde{Y}_i)(1-Sp_{(2)}))$. We generate 500 datasets for each scenario.

A tolerance threshold of $\epsilon = 10^{-3}$ for the change in the ELBO and a maximum of 50,000 iterations are used as the stopping criterion for the VB algorithm. The convergence of the VB algorithm is assessed by examining the stability of the estimators. For the MPT and DT scenarios, we assume a variational distribution for the $\tilde{Y}_i$s which allowed for dependence among the infection statuses of individuals tested in the same pool. Please see Web Appendix A.1 and B.1 for details.

We compare our proposed VB algorithm against a traditional MCMC approach for fitting Bayesian group testing regression models similar to that used in Huang et al. (2024). For the Bayesian group testing regression model, posterior distributions are sampled using the MCMC approach across simulation scenarios, including the MPT and IT protocols with known $\boldsymbol{Se}$ and $\boldsymbol{Sp}$ and the DT protocol with unknown $\boldsymbol{Se}$ and $\boldsymbol{Sp}$. For each parameter, we generate a chain of 5,000 MCMC samples with a burn-in of 10,000 draws. After discarding the burn-in, the posterior mean and posterior standard deviation are estimated using the mean and standard deviation of the retained MCMC sample; 95% equal-tailed credible intervals are constructed in the usual way using the sample quantiles of the posterior sample.

Web Appendix C.1 contains additional details about the initialization procedure for both algorithms.

To assess the estimation accuracy of the VB method, the average empirical bias of the VB mean estimates (VB Bias) is reported. To compare the inferential reliability of the traditional and hybrid VB inference approaches, we report the sample standard deviation of the 500 VB mean estimators (VB SSD), the average standard deviation of the optimal variational distribution (VB SD) and the average standard deviation of the hybrid inference approach (the square roots of the diagonal elements of $\Sigma _{q*}$, VB HSD). Additionally, we report empirical coverage probabilities for 95% credible intervals for the traditional VB approach (VB CP) and 95% Wald confidence intervals from the hybrid approach (VB HCP). For the MCMC algorithm, we report the average empirical bias of mean of the posterior sample (MCMC Bias), the sample standard deviation of the 500 posterior mean estimates (MCMC SSD) and the average standard deviations (MCMC SD) of the posterior sample. We also report the empirical coverage probabilities (MCMC CP) of 95% equal-tailed credible intervals for the MCMC algorithm.

Comparing the computation time for the two methods is somewhat nuanced, as the computation time depends on the stopping criteria for each algorithm. Specifically, the computation time for the VB algorithm depends on the specified tolerance threshold ϵ and the maximum allowable number of VB iterations while the computation time for the MCMC algorithm depends the total number of MCMC iterations (the burn-in period plus the posterior sample size). Fairly comparing the computational efficiency of the two algorithms requires addressing the potential trade-off between runtime and overall reliability. For the VB algorithm, using a smaller tolerance threshold for stopping potentially increases confidence in convergence, but also increases the runtime of the algorithm. Increasing the length of the burn-in period in the MCMC algorithm generally increases our confidence that the MCMC chain has converged, especially in scenarios were convergence diagnostics using shorter burn-in periods indicate lack of convergence; obtaining a larger posterior sample after convergence reduces the impact of Monte Carlo error. To fairly compare the tradeoff between run-time and estimation/inference for the two algorithms, we run both algorithms under a variety of different stopping criteria and plot the performance, measured by absolute parameter bias and empirical coverage probabilities, as a function of runtime. Please see Web Appendix C for more information.

### Simulation results

Tables 1, 2 and 3 summarize the results for the regression coefficients from the VB and MCMC methods under MPT, IT and DT, respectively. The VB algorithm exhibits smaller bias than the MCMC when the sample size is small (N=300,1500), but the two methods exhibit comparable bias for the larger sample sizes. Furthermore, in the N=300 MPT and DT scenarios, the bias from both methods is rather large. IT exhibits much better performance for the N=300 case, suggesting that individual testing may be preferred to pooled testing when the sample size is very small.

The standard deviation of the optimal VB distribution (VB SD) underestimates the true posterior standard deviation estimated by the standard deviation of the posterior sample obtained from MCMC (MCMC SD); as the optimal VB distribution is well-known to underestimate the uncertainty in the true posterior distribution, this result is not surprising. Empirical coverage probabilities for credible intervals derived from the optimal VB distribution (VB CP) fail to attain their nominal levels. We note that the standard deviation derived from $\Sigma _{q*}$ under the hybrid inference approach (VB HSD) is approximating the standard deviation of the *asymptotic sampling distribution* of $\boldsymbol{\hat{\theta }_{VB}}$, and thus we would expect VB HSD to agree with the observed sample standard deviation of the VB point estimators (VB SSD). We find that these two quantities agree closely for all scenarios except the N=300, pool size =10 MPT configuration, which has only 30 observed testing responses (likely not enough data for asymptotic results to be reliable). Notably, empirical coverage probabilities for the regression coefficients from the hybrid inference approach attain their nominal levels in all scenarios, even for the smallest sample sizes. We also find that the estimated posterior standard deviation from MCMC (MCMC SD) and the observed sample standard deviation of the MCMC posterior mean estimators (MCMC SSD) are closely aligned. Furthermore, these two quantities agree with VB HSD and VB SSD provided the sample size is at least 1500. However, credible intervals obtained from MCMC (MCMC CP) fail to attain their nominal level in the low-information scenarios (N=300, pool size =3,10, and N=1500, pool size = 10). While the MCMC algorithm is estimating the true posterior distribution, these credible intervals are guaranteed to attain their nominal levels only asymptotically (as a consequence of the Bernstein-von Mises theorem) and may not attain their nominal levels for small sample sizes even when the posterior distribution is reliably estimated.

Table 4 displays the results for $\boldsymbol{Se}$ and $\boldsymbol{Sp}$ from the DT scenarios. In general these results display the same trends as the results for the regression coefficients, though VB HSD and VB SSD require a slightly larger sample size (N=3000) to attain alignment.

**Table 1:** Simulation result of variational Bayes (VB) and Markov chain Monte Carlo (MCMC) method for group testing data under the master pool testing (MPT) protocol with known sensitivity and specificity

			VB						MCMC			
N	Pool size	Param	Bias	SSD	SD	CP	HSD	HCP	Bias	SSD	SD	CP
300	3	$\beta _0=$ −4	−0.521	1.157	0.243	0.392	1.048	0.974	−0.924	1.391	1.281	0.900
		$\beta _1=$ 2	0.244	0.667	0.166	0.408	0.605	0.970	0.421	0.774	0.685	0.904
		$\beta _2=$ 1	0.242	1.049	0.317	0.512	0.901	0.982	0.364	1.384	1.157	0.922
1500	3	$\beta _0=$ −4	−0.067	0.378	0.104	0.428	0.360	0.952	−0.141	0.375	0.372	0.948
		$\beta _1=$ 2	0.046	0.234	0.072	0.484	0.230	0.950	0.071	0.239	0.235	0.936
		$\beta _2=$ 1	0.002	0.332	0.137	0.596	0.329	0.960	0.074	0.326	0.336	0.950
3000	3	$\beta _0=$ −4	−0.020	0.253	0.074	0.422	0.250	0.952	−0.039	0.249	0.251	0.954
		$\beta _1=$ 2	0.011	0.157	0.050	0.472	0.160	0.954	0.028	0.162	0.161	0.938
		$\beta _2=$ 1	−0.006	0.234	0.097	0.572	0.230	0.948	−0.003	0.233	0.231	0.950
6000	3	$\beta _0=$ −4	−0.021	0.177	0.052	0.448	0.176	0.950	−0.043	0.186	0.176	0.922
		$\beta _1=$ 2	0.021	0.115	0.036	0.456	0.113	0.946	0.012	0.117	0.112	0.930
		$\beta _2=$ 1	−0.004	0.167	0.068	0.570	0.162	0.944	0.022	0.160	0.163	0.954
12,000	3	$\beta _0=$ −4	−0.015	0.124	0.037	0.430	0.124	0.958	−0.013	0.121	0.124	0.958
		$\beta _1=$ 2	0.007	0.075	0.025	0.468	0.079	0.966	0.007	0.083	0.079	0.934
		$\beta _2=$ 1	0.011	0.120	0.048	0.568	0.114	0.946	0.005	0.107	0.114	0.958
300	10	$\beta _0=$ −4	−1.408	2.380	0.260	0.212	3.409	0.942	−3.173	3.552	3.446	0.824
		$\beta _1=$ 2	0.592	2.018	0.177	0.218	1.546	0.916	1.355	3.074	2.244	0.822
		$\beta _2=$ 1	0.162	2.928	0.342	0.240	4.102	0.984	0.663	4.223	4.142	0.864
1500	10	$\beta _0=$ −4	−0.253	0.770	0.106	0.252	0.701	0.948	−0.418	1.718	0.939	0.894
		$\beta _1=$ 2	0.121	0.503	0.073	0.256	0.450	0.952	0.053	1.364	0.532	0.896
		$\beta _2=$ 1	0.118	0.708	0.139	0.328	0.681	0.958	0.454	1.525	0.984	0.902
3000	10	$\beta _0=$ −4	−0.137	0.457	0.074	0.262	0.464	0.972	−0.155	0.880	0.494	0.934
		$\beta _1=$ 2	0.066	0.294	0.051	0.268	0.302	0.960	0.053	0.656	0.313	0.920
		$\beta _2=$ 1	0.084	0.477	0.097	0.304	0.459	0.962	0.088	0.665	0.506	0.950
6000	10	$\beta _0=$ −4	−0.054	0.328	0.052	0.274	0.316	0.960	−0.091	0.314	0.317	0.942
		$\beta _1=$ 2	0.030	0.220	0.036	0.238	0.209	0.938	0.061	0.209	0.210	0.940
		$\beta _2=$ 1	0.023	0.300	0.068	0.374	0.317	0.966	0.019	0.326	0.318	0.942
12,000	10	$\beta _0=$ −4	−0.025	0.212	0.037	0.264	0.221	0.954	−0.051	0.221	0.222	0.936
		$\beta _1=$ 2	0.004	0.138	0.025	0.276	0.146	0.960	0.024	0.147	0.146	0.952
		$\beta _2=$ 1	0.018	0.219	0.048	0.360	0.222	0.956	0.030	0.224	0.222	0.954

**Table 2:** Simulation result of variational Bayes (VB) and Markov chain Monte Carlo (MCMC) method for group testing data under the individual testing (IT) protocol with known sensitivity and specificity

			VB						MCMC			
N	Pool size	Param	Bias	SSD	SD	CP	HSD	HCP	Bias	SSD	SD	CP
300	1	$\beta _0=$ −4	−0.265	0.771	0.238	0.474	0.717	0.956	−0.523	0.953	0.809	0.918
		$\beta _1=$ 2	0.117	0.478	0.163	0.568	0.436	0.942	0.294	0.557	0.483	0.918
		$\beta _2=$ 1	0.108	0.570	0.312	0.750	0.573	0.962	0.145	0.708	0.623	0.930
1500	1	$\beta _0=$ −4	−0.035	0.298	0.104	0.486	0.284	0.952	−0.073	0.298	0.289	0.948
		$\beta _1=$ 2	0.018	0.186	0.071	0.574	0.176	0.946	0.047	0.193	0.180	0.928
		$\beta _2=$ 1	0.019	0.237	0.137	0.738	0.237	0.950	0.016	0.237	0.239	0.930
3000	1	$\beta _0=$ −4	−0.030	0.208	0.074	0.498	0.200	0.946	−0.058	0.207	0.202	0.940
		$\beta _1=$ 2	0.020	0.128	0.050	0.584	0.124	0.942	0.031	0.127	0.125	0.936
		$\beta _2=$ 1	0.001	0.170	0.097	0.740	0.167	0.944	0.016	0.165	0.168	0.950
6000	1	$\beta _0=$ −4	−0.016	0.145	0.052	0.522	0.140	0.938	−0.026	0.139	0.141	0.954
		$\beta _1=$ 2	0.007	0.091	0.036	0.552	0.087	0.946	0.014	0.087	0.087	0.956
		$\beta _2=$ 1	0.009	0.122	0.068	0.754	0.118	0.930	0.016	0.116	0.118	0.952
12,000	1	$\beta _0=$ −4	−0.007	0.098	0.037	0.566	0.099	0.940	−0.020	0.102	0.099	0.944
		$\beta _1=$ 2	0.002	0.062	0.025	0.580	0.061	0.946	0.010	0.064	0.061	0.936
		$\beta _2=$ 1	0.004	0.081	0.048	0.766	0.083	0.954	0.009	0.087	0.083	0.940

**Table 3:** Simulation results for the regression coefficients from the variational Bayes (VB) and Markov chain Monte Carlo (MCMC) methods for group testing data under the Dorfman testing (DT) protocol with unknown sensitivity and specificity

			VB						MCMC			
N	Pool size	Param	Bias	SSD	SD	CP	HSD	HCP	Bias	SSD	SD	CP
300	3	$\beta _0=$ −4	−0.496	1.078	0.243	0.448	1.080	0.966	−1.531	1.864	1.723	0.880
		$\beta _1=$ 2	0.550	0.793	0.169	0.386	0.837	0.956	1.623	1.581	1.456	0.736
		$\beta _2=$ 1	0.258	0.774	0.315	0.630	0.782	0.958	0.694	1.368	1.174	0.898
1500	3	$\beta _0=$ −4	−0.065	0.295	0.104	0.508	0.305	0.962	−0.087	0.313	0.299	0.936
		$\beta _1=$ 2	0.111	0.201	0.072	0.496	0.205	0.950	0.149	0.190	0.203	0.934
		$\beta _2=$ 1	0.076	0.257	0.137	0.706	0.251	0.952	0.078	0.261	0.253	0.936
3000	3	$\beta _0=$ −4	−0.032	0.195	0.074	0.558	0.205	0.956	−0.035	0.186	0.199	0.958
		$\beta _1=$ 2	0.065	0.128	0.051	0.548	0.139	0.964	0.075	0.128	0.135	0.936
		$\beta _2=$ 1	0.032	0.170	0.097	0.750	0.172	0.942	0.052	0.167	0.172	0.944
6000	3	$\beta _0=$ −4	−0.013	0.140	0.052	0.552	0.141	0.952	0.001	0.141	0.137	0.940
		$\beta _1=$ 2	0.036	0.088	0.036	0.564	0.097	0.960	0.026	0.089	0.092	0.944
		$\beta _2=$ 1	0.019	0.123	0.068	0.704	0.120	0.948	0.012	0.116	0.119	0.962
12,000	3	$\beta _0=$ −4	0.003	0.098	0.037	0.552	0.098	0.954	−0.003	0.099	0.097	0.942
		$\beta _1=$ 2	0.020	0.066	0.025	0.528	0.068	0.940	0.014	0.065	0.065	0.954
		$\beta _2=$ 1	0.004	0.082	0.048	0.744	0.084	0.960	0.003	0.081	0.083	0.960
300	10	$\beta _0=$ −4	−0.936	1.599	0.251	0.344	1.961	0.962	−3.105	2.363	2.816	0.782
		$\beta _1=$ 2	0.814	1.250	0.174	0.334	1.470	0.918	3.039	2.360	2.467	0.622
		$\beta _2=$ 1	0.494	1.039	0.322	0.564	1.144	0.944	1.148	1.863	1.777	0.866
1500	10	$\beta _0=$ −4	−0.107	0.320	0.105	0.474	0.353	0.970	−0.128	0.355	0.341	0.926
		$\beta _1=$ 2	0.109	0.206	0.072	0.496	0.255	0.960	0.137	0.230	0.220	0.902
		$\beta _2=$ 1	0.063	0.252	0.137	0.706	0.266	0.960	0.058	0.255	0.260	0.944
3000	10	$\beta _0=$ −4	−0.043	0.227	0.074	0.458	0.230	0.954	−0.071	0.220	0.227	0.952
		$\beta _1=$ 2	0.050	0.136	0.051	0.534	0.149	0.960	0.070	0.141	0.142	0.926
		$\beta _2=$ 1	0.014	0.182	0.097	0.706	0.176	0.946	0.032	0.174	0.176	0.956
6000	10	$\beta _0=$ −4	−0.032	0.159	0.052	0.478	0.159	0.960	−0.020	0.166	0.157	0.942
		$\beta _1=$ 2	0.026	0.099	0.036	0.500	0.100	0.948	0.024	0.099	0.098	0.948
		$\beta _2=$ 1	0.030	0.121	0.068	0.706	0.123	0.970	0.005	0.124	0.122	0.948
12,000	10	$\beta _0=$ −4	−0.013	0.107	0.037	0.482	0.111	0.962	−0.026	0.110	0.110	0.948
		$\beta _1=$ 2	0.013	0.067	0.025	0.540	0.069	0.950	0.018	0.070	0.068	0.938
		$\beta _2=$ 1	0.007	0.081	0.048	0.750	0.086	0.962	0.010	0.088	0.086	0.954

**Table 4:** Simulation results for the assay accuracy parameters from the variational Bayes (VB) and Markov chain Monte Carlo (MCMC) methods for group testing data under the Dorfman testing (DT) protocol with unknown sensitivity and specificity

			VB						MCMC			
N	Pool size	Param	Bias	SSD	SD	CP	HSD	HCP	Bias	SSD	SD	CP
300	3	$Se_{(1)}=$ 0.95	−0.149	0.108	0.065	0.408	0.221	0.890	−0.218	0.116	0.129	0.738
		$Se_{(2)}=$ 0.98	−0.056	0.041	0.047	0.768	0.178	0.992	−0.025	0.025	0.030	0.942
		$Sp_{(1)}=$ 0.98	−0.014	0.018	0.021	0.938	0.086	0.992	−0.081	0.051	0.070	0.880
		$Sp_{(2)}=$ 0.99	−0.033	0.025	0.027	0.738	0.111	0.982	−0.048	0.034	0.039	0.834
1500	3	$Se_{(1)}=$ 0.95	−0.057	0.056	0.024	0.488	0.101	0.960	−0.068	0.050	0.068	0.946
		$Se_{(2)}=$ 0.98	−0.016	0.019	0.015	0.772	0.041	0.998	−0.002	0.009	0.010	0.970
		$Sp_{(1)}=$ 0.98	−0.001	0.009	0.007	0.906	0.012	1.000	−0.020	0.017	0.024	0.944
		$Sp_{(2)}=$ 0.99	−0.007	0.009	0.008	0.838	0.018	0.996	−0.009	0.010	0.011	0.944
3000	3	$Se_{(1)}=$ 0.95	−0.034	0.046	0.016	0.484	0.071	0.964	−0.041	0.040	0.051	0.962
		$Se_{(2)}=$ 0.98	−0.009	0.015	0.010	0.748	0.022	0.978	0.000	0.006	0.007	0.974
		$Sp_{(1)}=$ 0.98	0.000	0.006	0.005	0.878	0.008	0.996	−0.011	0.014	0.017	0.956
		$Sp_{(2)}=$ 0.99	−0.003	0.007	0.005	0.820	0.010	0.996	−0.004	0.006	0.007	0.966
6000	3	$Se_{(1)}=$ 0.95	−0.021	0.034	0.011	0.422	0.050	0.966	−0.021	0.029	0.038	0.976
		$Se_{(2)}=$ 0.98	−0.004	0.010	0.006	0.730	0.014	0.988	0.000	0.005	0.005	0.950
		$Sp_{(1)}=$ 0.98	0.000	0.004	0.004	0.888	0.005	0.966	−0.005	0.011	0.012	0.956
		$Sp_{(2)}=$ 0.99	−0.002	0.005	0.003	0.802	0.006	0.990	−0.001	0.005	0.005	0.958
12,000	3	$Se_{(1)}=$ 0.95	−0.014	0.028	0.007	0.372	0.035	0.954	−0.008	0.024	0.029	0.974
		$Se_{(2)}=$ 0.98	−0.002	0.008	0.004	0.686	0.009	0.980	0.000	0.003	0.003	0.940
		$Sp_{(1)}=$ 0.98	0.000	0.003	0.003	0.854	0.004	0.950	−0.002	0.008	0.008	0.946
		$Sp_{(2)}=$ 0.99	−0.001	0.004	0.002	0.756	0.004	0.968	0.000	0.004	0.004	0.968
300	10	$Se_{(1)}=$ 0.95	−0.118	0.088	0.072	0.610	0.239	0.878	−0.164	0.094	0.105	0.736
		$Se_{(2)}=$ 0.98	−0.102	0.083	0.055	0.552	0.256	0.934	−0.194	0.103	0.160	0.878
		$Sp_{(1)}=$ 0.98	−0.123	0.073	0.103	0.800	0.535	0.998	−0.207	0.107	0.129	0.726
		$Sp_{(2)}=$ 0.99	−0.018	0.017	0.012	0.680	0.046	0.928	−0.027	0.017	0.021	0.756
1500	10	$Se_{(1)}=$ 0.95	−0.033	0.041	0.026	0.682	0.086	0.976	−0.038	0.036	0.049	0.966
		$Se_{(2)}=$ 0.98	−0.016	0.020	0.015	0.792	0.090	1.000	−0.025	0.018	0.034	0.996
		$Sp_{(1)}=$ 0.98	−0.023	0.022	0.027	0.906	0.134	1.000	−0.025	0.020	0.033	0.982
		$Sp_{(2)}=$ 0.99	−0.003	0.006	0.004	0.746	0.009	0.976	−0.004	0.007	0.007	0.942
3000	10	$Se_{(1)}=$ 0.95	−0.019	0.034	0.017	0.628	0.047	0.968	−0.018	0.031	0.035	0.960
		$Se_{(2)}=$ 0.98	−0.009	0.015	0.010	0.782	0.044	1.000	−0.012	0.013	0.022	1.000
		$Sp_{(1)}=$ 0.98	−0.011	0.015	0.016	0.918	0.054	1.000	−0.011	0.013	0.021	0.994
		$Sp_{(2)}=$ 0.99	−0.001	0.005	0.003	0.692	0.005	0.974	−0.002	0.005	0.005	0.948
6000	10	$Se_{(1)}=$ 0.95	−0.011	0.025	0.012	0.546	0.031	0.972	−0.010	0.024	0.026	0.966
		$Se_{(2)}=$ 0.98	−0.002	0.011	0.006	0.654	0.025	1.000	−0.006	0.011	0.015	0.998
		$Sp_{(1)}=$ 0.98	−0.006	0.012	0.011	0.886	0.028	1.000	−0.004	0.010	0.015	0.992
		$Sp_{(2)}=$ 0.99	−0.001	0.003	0.002	0.700	0.003	0.970	−0.001	0.003	0.003	0.946
12,000	10	$Se_{(1)}=$ 0.95	−0.005	0.019	0.008	0.582	0.021	0.974	−0.003	0.019	0.019	0.960
		$Se_{(2)}=$ 0.98	−0.002	0.010	0.004	0.574	0.015	1.000	−0.003	0.009	0.012	0.988
		$Sp_{(1)}=$ 0.98	−0.003	0.010	0.007	0.782	0.017	1.000	−0.002	0.009	0.011	0.982
		$Sp_{(2)}=$ 0.99	0.000	0.002	0.001	0.702	0.002	0.932	−0.001	0.002	0.002	0.946

Web Figures C.1–C.6 compare the relative performance of the VB and MCMC algorithms as a function of runtime. Web Figures C.1–C.2 show the maximum empirical absolute bias of the three $\boldsymbol{\beta }$ coefficients from the VB algorithm fit using a decreasing sequence of tolerance thresholds for the ELBO. In most scenarios, the empirical absolute bias stabilizes at a relatively liberal tolerance threshold ($\epsilon \approx 0.01$); using stricter stopping criteria for the VB algorithm increases the runtime but does not improve performance. The same figures also show the maximum empirical absolute bias of the three regression coefficients from the MCMC algorithm with varying burn-in periods. The absolute bias of the MCMC algorithm also stabilizes quickly, but in many scenarios it stabilizes at a higher value than the absolute bias of the VB algorithm. Figures C.3–C.4 display the minimum empirical coverage probability (MECP) of the regression coefficients from the VB hybrid inference and MCMC approaches under the same conditions as Figures C.1–C.2. The MECPs from the hybrid inference approach stabilize quickly at their nominal level and show little change as the tolerance threshold continues to decrease. The MECPs from the MCMC algorithm are more variable. In several of the smaller sample size scenarios, the MCMC MECPs never attain their nominal level, even allowing for the expected margin of Monte Carlo error. In contrast, the MECPs from the VB hybrid inference approach stabilize within the Monte Carlo margin of error of the nominal nominal level in all scenarios. Figures C.5–C.6 contain analogous results for the assay accuracy parameters from the DT scenarios and exhibit similar overall patterns.

Our simulation study illustrates the VB algorithm has several advantages over the MCMC algorithm. First, in many instances, the VB approach provides lower bias estimators than the MCMC approach, especially when the sample size is small. Second, the VB hybrid inference approach is reliable in small sample size, large pool size scenarios for which credible intervals from full MCMC inference fail to sufficiently capture the true parameter values. The performance of the VB hybrid inference approach is also robust to the choice of stopping criteria for the algorithm, while the performance of the MCMC algorithm is varies more with the choice of stopping criteria; for a real data application it can be difficult to determine how long a burn-in period and how large a posterior sample size is necessary for reliable MCMC inference, necessitating the use of a long burn-in period and large sample size at the cost of additional runtime. In contrast, even a liberal ELBO convergence threshold of 0.01 produces reliable VB hybrid inference in all scenarios and inferential reliability does not vary with the VB stopping criteria. Finally, in most scenarios, the VB algorithm requires less computation time to attain the ‘point of reliability’ than the MCMC algorithm.

## Data application

The National Institutes of Health’s All of Us research program is a large biomedical data resource, containing health data from a diverse group of participants across the United States (National Institutes of Health 2024). In this project, we apply the proposed VB and MCMC methods to the participant-level COVID-19 RNA laboratory testing data and demographic survey responses collected between 2019 and 2020. After processing the data by restricting it to the target time window and combining low-frequency categories into the categories with reasonable counts for analysis, the final analytical dataset contains laboratory results and survey responses from N=43,185 individuals. The overall positivity rate of COVID-19 RNA laboratory results is around 8%. Covariates includes participant’s age, race/ethnicity, education level, and general health conditions. Web Appendix Table C.1 summarizes frequency counts for the demographic characteristics of this study population.

To fit our group testing models, we simulate pooled testing responses from the individual testing results. This approach allows us to compare different group testing protocols. Under the MPT and DT protocols, individuals are placed in pools of size 3 and the pool test result is taken to be the maximum of individual test results of the contributors. The original COVID-19 laboratory test results for individuals in the positive pools are retained as individual retesting information under the DT protocol. For comparison, these original test results are also used as individual pool responses under the IT protocol.

First, we apply the VB method under the DT protocol with unknown testing accuracy probabilities to estimate the sensitivity and specificity of testing. The initial values for these parameters are selected based on a systematic review of diagnosis accuracy for similar COVID-19 RNA tests (Mustafa Hellou et al. 2021). Once the estimates for sensitivity and specificity are obtained, we treat them as known values when applying the MPT and IT protocols. For comparison, the MCMC algorithm is also applied under the all scenarios. Both algorithms are implemented in the same manner as in the simulation study. That is, the VB algorithm is implemented with an ELBO tolerance threshold of $\epsilon = 10^{-3}$ and using a variational factorization which allows for dependence between the $\tilde{Y}_i$s for individuals in the same pool. The MCMC algorithm used a burn-in period of 10,000 iterations and a posterior sample size of 5000 iterations and convergence was assessed with Geweke diagnostics. As $\boldsymbol{Se}$ and $\boldsymbol{Sp}$ are weakly identifiably under MPT and IT, we first fit the model using DT to estimate $\boldsymbol{Se}$ and $\boldsymbol{Sp}$. We then fixed $\boldsymbol{Se}$ and $\boldsymbol{Sp}$ at the posterior mean estimate obtained from the DT model when fitting the MPT and IT comparison models.

The means of the optimal variational distribution (Estimate), standard deviations of the optimal variational distribution (SD), 95% credible intervals using traditional VB inference (95% CI), standard deviations derived from $\Sigma _{q^*}$ (HSD) and associated 95% Wald confidence intervals (95% HCI) are reported for the VB approach, along with mean of the posterior sample (Estimate), standard deviation of the posterior sample (SD) and 95% equal-tailed credible intervals (95% CI) for the MCMC approach. These results are reported in Table 5, Appendix Tables C.2 and C.3 for DT, MPT and IT, respectively. Additionally, computation time is recorded for each method.

**Table 5:** Results of variational Bayesian (VB) and Markov chain Monte Carlo (MCMC) method under the Dorfman testing protocol with unknown sensitivity and specificity for 2019–2020 All of Us Research Program COVID data

	VB method					MCMC algorithm		
Parameter	Estimate	SD	95% CI	HSD	95% HCI	Estimate	SD	95% CI
(Intercept)	−2.611	0.024	( −2.658, −2.564 )	0.016	( −2.643, −2.579 )	−2.604	0.047	( −2.693, −2.506 )
Age	−0.105	0.012	( −0.128, −0.082 )	0.018	( −0.140, −0.070 )	−0.104	0.018	( −0.140, −0.068 )
Race/ethnicity								
Black	0.045	0.033	( −0.019, 0.110 )	0.050	( −0.054, 0.144 )	0.046	0.050	( −0.052, 0.146 )
Hispanic	0.361	0.033	( 0.296, 0.426 )	0.048	( 0.268, 0.455 )	0.363	0.048	( 0.266, 0.457 )
Other races/	−0.058	0.045	( −0.147, 0.030 )	0.072	( −0.200, 0.084 )	−0.059	0.072	( −0.201, 0.077 )
unknown/declined								
White	ref	ref	ref	ref	ref	ref	ref	ref
Education								
College one to three	0.366	0.030	( 0.308, 0.424 )	0.047	( 0.274, 0.457 )	0.365	0.046	( 0.277, 0.455 )
High school or GED	0.409	0.033	( 0.344, 0.475 )	0.051	( 0.308, 0.510 )	0.411	0.052	( 0.308, 0.515 )
Less than high school	0.536	0.045	( 0.448, 0.624 )	0.066	( 0.407, 0.664 )	0.536	0.065	( 0.410, 0.661 )
Unknown/declined	0.296	0.071	( 0.158, 0.435 )	0.109	( 0.083, 0.510 )	0.294	0.110	( 0.075, 0.510 )
College and above	ref	ref	ref	ref	ref	ref	ref	ref
General health								
Good or fair	−0.153	0.026	( −0.204, −0.103 )	0.039	( −0.230, −0.077 )	−0.153	0.038	( −0.229, −0.077 )
Poor	−0.470	0.050	( −0.567, −0.373 )	0.080	( −0.626, −0.313 )	−0.474	0.079	( −0.633, −0.321 )
Unknown/declined	−0.041	0.092	( −0.221, 0.139 )	0.137	( −0.310, 0.228 )	−0.046	0.137	( −0.324, 0.221 )
Excellent or very good	ref	ref	ref	ref	ref	ref	ref	ref
Pool Se	0.9734	7.6 $\times 10^{-7}$	( 0.97334146, 0.97337150 )	0.0386	( 0.9676711, 0.9785121 )	0.9653	0.0238	( 0.90288028, 0.99656263 )
Individual Se	0.9997	8.7 $\times 10^{-8}$	( 0.99968932, 0.99968967 )	0.0190	( 0.9989050, 0.9999892 )	0.9999	0.0001	( 0.99967046, 0.99999777 )
Pool Sp	0.9999	8.0 $\times 10^{-9}$	( 0.99990895, 0.99990898 )	0.0045	( 0.9996646, 0.9999977 )	0.9997	0.0003	( 0.99876733, 0.99999212 )
Individual Sp	0.9983	2.7 $\times 10^{-7}$	( 0.99827992, 0.99828099 )	0.0041	( 0.9971170, 0.9991464 )	0.9980	0.0017	( 0.99386944, 0.99993527 )

The VB and MCMC methods produce similar parameter estimates. The standard deviations and confidence intervals from the hybrid VB method are similar to the posterior standard deviations and credible intervals from the MCMC approach, while the traditional VB inference approach tends to produce smaller standard deviations. As the group testing data was generated by directly combining individual testing results without access to actual retesting information, it is challenging to estimate the sensitivity and specificity for individual testing. Additionally, using the synthetic pools also impacts the interpretation of the sensitivity and specificity estimates under MPT and DT. These limitations affect both the VB and MCMC methods.

In terms of computation time, consistent with findings from the simulation study, the VB algorithm is generally faster than the MCMC algorithm. The VB algorithm took 7.5 min for the DT protocol, 8.0 s for the MPT protocol, and 6.9 s for the IT protocol, while the MCMC algorithm took 11.1 min for DT, 11.2 min for MPT and 12.2 min for IT.

As a sensitivity analysis, the VB algorithm was run using a tolerance threshold of $\epsilon = 10^{-4}$ for the DT, MPT and IT protocols. Parameters estimates from the sensitivity analysis were identical to those from the primary analysis to four decimal places.

## Conclusion

In this study, we developed and formalized variational Bayesian inference for group testing data across different group testing protocols. Furthermore, we introduced a hybrid inference approach to overcome the shortcomings of traditional VB inference by using the asymptotic normality property of the VB mean estimators. In a rigorous simulation study and data application, the hybrid VB inference method demonstrated almost identical performance to full MCMC-based inference, with the added advantage of faster computation, especially under relatively large sample scenarios. The hybrid VB method retained its computational advantage when applied to COVID-19 testing data from the NIH’s All of Us research program.

Our methodology suggests a number of areas for future work. While this work focuses exclusively on the case of the logistic link function, group testing regression models have also been developed using other link functions, most notably the probit link (Joyner et al. 2020; Mcmahan et al. 2025). The development of a variational Bayesian inference framework for group testing probit regression, perhaps by extending Consonni and Marin (2007)’s method for variational inference for probit regression (based on the data augmentation approach of Albert and Chib (1993)) is an excellent area for future work. Extensions to more complicated Bayesian group testing models, including mixed effects models (Joyner et al. 2020), spatial models (Huang et al. 2024), and non-linear models (St. Ville, M.E., McMahan, C.S., Bible, J.D., Tebbs, J.M., Bilder, C.R., 2025) are highly desirable. An exploration of the theoretical properties of the hybrid inference approach and the development of technical conditions under which $\Sigma _{q^*}$ is a good approximation the asymptotic covariance matrix of the VB mean estimators is also of interest. Such an investigation need not be limited to the group testing context, but could consider VB models more broadly. As the size and complexity of biomedical datasets continues to grow, so will the need for scalable inference.

## Supplementary Information

Below is the link to the electronic supplementary material.

Supplementary file 1 (pdf 14918 KB)

## Data Availability

The data used in this study is available upon request from the National Institutes of Health All of Us Research Program: https://allofus.nih.gov/.

## Declarations

### AI technologies in the manuscript preparation process

During the preparation of this work the authors used AI technology in order to summarize some articles cited in the introduction and for language editing (e.g. spelling, grammar, and clarity). After using the AI tools, the authors reviewed and edited the content as needed and take full responsibility for the content of the published article. AI was also used to convert code previously written in R to C++ to leverage the Rcpp package. This code was then thoroughly tested by the authors.
