# Supplementary material for: Variational Bayesian inference for logistic regression models for group testing data: Comparing a new hybrid variational inference approach to traditional methods
Source: Stat Comput. 2026 Nov 5;36(6):256. doi: 10.1007/s11222-026-11013-5 (PMC13739965; doi:10.1007/s11222-026-11013-5)
Supplement: Supplementary file 1 [file 11222_2026_11013_MOESM1_ESM.pdf]

Supplementary Material for "Variational Bayesian Inference for Logistic Regression Models for Group Testing Data: Comparing a New Hybrid Variational Inference Approach to Traditional Methods"

Xingpei Zhao[a], Alexander McLain[a], Bo Cai[a], Dewei Wang[b], Stella Self[a]

[a] *Department of Epidemiology and Biostatistics, University of South Carolina, 915 Greene St, Columbia, 29208, South Carolina, United States*
[b] *Department of Statistics, University of South Carolina, 1523 Greene Street, Columbia, 29208, South Carolina, United States*

Keywords: Variational Bayes, Markov chain Monte Carlo, Pooled testing, Binary outcomes

Appendix A. Derivations of Variational Factors

In this section, we provide expressions for the optimal variational factor $q^{(t)}(\tilde{Y}_i)$ under three testing protocols: Dorfman testing (DT), master pool testing (MPT) and individual testing (IT). Recall that under MPT, each individual is tested in exactly one pool and no individuals are retested. DT is similar to MPT, but contributors to positive pools are individually retested. IT is equivalent to traditional testing, in which each individual is tested in a 'pool' of size one. For notational simplicity, we assume a universal pool size of 3 for the DT and MPT protocols, though this approach can easily to adapted to accomodate other pool sizes.

Appendix A.1. Derivation the optimal variational factor for $\tilde{\boldsymbol{Y}}$

Under the Dorfman Testing (DT) protocol with a fixed pool size of 3. Here, we relax the assumption that $q(\tilde{\boldsymbol{Y}}) = \prod_{i=1}^{N} q(\tilde{Y}_i)$ and instead assume that $q(\tilde{\boldsymbol{Y}}) = \prod_{i=1}^{J^*} q(\tilde{Y}_i : i \in \mathcal{P}_j)$, where $J^* = N/3$ is the number of 'original' pools tested in the first stage. Thus we are factoring $q(\tilde{\boldsymbol{Y}})$ over pools rather than individuals, allowing for dependence between individuals in the same pool.

We assume individuals l, m, and n are assigned pool j, so that $\mathcal{P}_j = \{l, m, n\}$, and the corresponding 'retest' pools for individual l, m, n are indexed by l^*, m^*, n^*, if such pools exist. We use $Z_{l^*}, Z_{m^*}, Z_{n^*}$ to represent the observed test responses from these retest pools and allow $Se_{l^*}, \ldots, Sp_{m^*}$ to denote corresponding sensitivity and specificity.

The optimal variational factor is given by

$$q^{(t)}\left(\tilde{Y}_l, \tilde{Y}_m, \tilde{Y}_n\right) \propto \exp\left\{\mathbb{E}_{\boldsymbol{\beta},\boldsymbol{\omega},\boldsymbol{Se},\boldsymbol{Sp}} \log p\left(\tilde{Y}_l, \tilde{Y}_m, \tilde{Y}_n \mid \boldsymbol{Z}, \boldsymbol{\beta}, \omega, \boldsymbol{Se}, \boldsymbol{Sp}\right)\right\}.$$

Note that

$$\Pr\left(\tilde{Y}_l = \tilde{y}_l, \tilde{Y}_m = \tilde{y}_m, \tilde{Y}_n = \tilde{y}_n \mid \boldsymbol{Z}, \boldsymbol{\beta}, \boldsymbol{\omega}, \boldsymbol{Se}, \boldsymbol{Sp}\right) = \frac{p_{\tilde{y}_l,\tilde{y}_m,\tilde{y}_n}}{\sum_{x=0}^{1}\sum_{y=0}^{1}\sum_{z=0}^{1} p_{xyz}}$$

for $\tilde{y}_l, \tilde{y}_m, \tilde{y}_n \in \{0, 1\}$, where the specific expressions for the $p_{\tilde{y}_l,\tilde{y}_m,\tilde{y}_n}$s depend on whether Z_j is 0 or 1.

When the corresponding pool response $Z_j = 0$, we have

$$\begin{aligned} p_{\tilde{y}_l,\tilde{y}_m,\tilde{y}_n} =& (1 - Se_j)^{\mathbb{I}\left(\sum_{i\in\mathcal{P}_j} \tilde{y}_i > 0\right)} Sp_j^{1-\mathbb{I}\left(\sum_{i\in\mathcal{P}_j} \tilde{y}_i > 0\right)} \\ &\times \prod_{i\in\mathcal{P}_j} g^{-1}(\eta_i)^{\tilde{y}_i}\left[1 - g^{-1}(\eta_i)\right]^{1-\tilde{y}_i}. \end{aligned}$$

When the corresponding pool response $Z_j = 1$, we have

$$\begin{aligned} p_{\tilde{y}_l,\tilde{y}_m,\tilde{y}_n} =& Se_j^{\mathbb{I}\left(\sum_{i\in\mathcal{P}_j} \tilde{y}_i > 0\right)} (1 - Sp_j)^{1-\mathbb{I}\left(\sum_{i\in\mathcal{P}_j} \tilde{y}_i > 0\right)} \\ &\times \prod_{i\in\mathcal{P}_j} \Bigg\{ \left[Se_{i^*}^{Z_{i^*}} (1 - Se_{i^*})^{1-Z_{i^*}}\right]^{\mathbb{I}(\tilde{y}_i > 0)} \\ &\qquad \left[Sp_{i^*}^{1-Z_{i^*}} (1 - Sp_{i^*})^{Z_{i^*}}\right]^{1-\mathbb{I}(\tilde{y}_i > 0)} \\ &\qquad g^{-1}(\eta_i)^{\tilde{y}_i}\left[1 - g^{-1}(\eta_i)\right]^{1-\tilde{y}_i} \Bigg\}. \end{aligned}$$

The optimal variational factor $q^{(t)}(\tilde{Y}_l, \tilde{Y}_m, \tilde{Y}_n)$ is provided below for each case.

When $Z_j = 0$,

$$
\begin{aligned}
q^{(t)}\left(\tilde{Y}_l, \tilde{Y}_m, \tilde{Y}_n\right) &\propto \exp\left\{\mathbb{E}_{\boldsymbol{\beta},\boldsymbol{\omega},\boldsymbol{Se},\boldsymbol{Sp}}\left[\log p\left(\tilde{Y}_l, \tilde{Y}_m, \tilde{Y}_n \mid Z_j = 0, \boldsymbol{\beta}, \boldsymbol{\omega}, \boldsymbol{Se}, \boldsymbol{Sp}\right)\right]\right\} \\
&\propto \exp\left\{\mathbb{E}_{\boldsymbol{\beta},\boldsymbol{\omega},\boldsymbol{Se},\boldsymbol{Sp}}\left[\log \frac{p_{\tilde{y}_l,\tilde{y}_m,\tilde{y}_n}}{\prod_{i\in\mathcal{P}_j}[1-g^{-1}(\eta_i)]}\right]\right\} \\
&\propto \exp\left\{\mathbb{E}_{\boldsymbol{\beta},\boldsymbol{\omega},\boldsymbol{Se},\boldsymbol{Sp}}\left[\log\left((1-Se_j)^{\mathbb{I}\left(\sum_{i\in\mathcal{P}_j}\tilde{Y}_i>0\right)} Sp_j^{1-\mathbb{I}\left(\sum_{i\in\mathcal{P}_j}\tilde{Y}_i>0\right)}\right)\right.\right. \\
&\qquad\qquad \left.\left. + \sum_{i\in\mathcal{P}_j}\tilde{Y}_i \log\left[\frac{g^{-1}(\eta_i)}{1-g^{-1}(\eta_i)}\right]\right]\right\} \\
&\propto \exp\left\{\mathbb{I}\left(\sum_{i\in\mathcal{P}_j}\tilde{Y}_i > 0\right)\mathbb{E}_{\boldsymbol{\beta},\boldsymbol{\omega},\boldsymbol{Se},\boldsymbol{Sp}}[\log(1-Se_j)]\right. \\
&\qquad \left. + \left[1-\mathbb{I}\left(\sum_{i\in\mathcal{P}_j}\tilde{Y}_i > 0\right)\right]\mathbb{E}_{\boldsymbol{\beta},\boldsymbol{\omega},\boldsymbol{Se},\boldsymbol{Sp}}(\log Sp_j) + \sum_{i\in\mathcal{P}_j}\tilde{Y}_i\,\mathbb{E}_{\boldsymbol{\beta},\boldsymbol{\omega},\boldsymbol{Se},\boldsymbol{Sp}}(\eta_i)\right\}.
\end{aligned}
$$

When $Z_j = 1$,

$$
\begin{aligned}
q^{(t)}\left(\tilde{Y}_l, \tilde{Y}_m, \tilde{Y}_n\right) &\propto \exp\left\{\mathbb{E}_{\boldsymbol{\beta},\boldsymbol{\omega},\boldsymbol{Se},\boldsymbol{Sp}}\left[\log p\left(\tilde{Y}_l, \tilde{Y}_m, \tilde{Y}_n \mid Z_j = 1, Z_{l^*}, Z_{m^*}, Z_{n^*}, \boldsymbol{\beta}, \boldsymbol{\omega}, \boldsymbol{Se}, \boldsymbol{Sp}\right)\right]\right\} \\
&\propto \exp\left\{\mathbb{E}_{\boldsymbol{\beta},\boldsymbol{\omega},\boldsymbol{Se},\boldsymbol{Sp}}\left[\log \frac{p_{\tilde{y}_l,\tilde{y}_m,\tilde{y}_n}}{\prod_{i\in\mathcal{P}_j}[1-g^{-1}(\eta_i)]}\right]\right\} \\
&\propto \exp\left\{\mathbb{I}\left(\sum_{i\in\mathcal{P}_j}\tilde{Y}_i > 0\right)\mathbb{E}_{\boldsymbol{\beta},\boldsymbol{\omega},\boldsymbol{Se},\boldsymbol{Sp}}(\log Se_j)\right. \\
&\qquad + \left[1-\mathbb{I}\left(\sum_{i\in\mathcal{P}_j}\tilde{Y}_i > 0\right)\right]\mathbb{E}_{\boldsymbol{\beta},\boldsymbol{\omega},\boldsymbol{Se},\boldsymbol{Sp}}[\log(1-Sp_j)] \\
&\qquad + \sum_{i\in\mathcal{P}_j}\left\{\tilde{Y}_i\left[Z_{i^*}\,\mathbb{E}_{\boldsymbol{\beta},\boldsymbol{\omega},\boldsymbol{Se},\boldsymbol{Sp}}(\log Se_{i^*}) + (1-Z_{i^*})\,\mathbb{E}_{\boldsymbol{\beta},\boldsymbol{\omega},\boldsymbol{Se},\boldsymbol{Sp}}[\log(1-Se_{i^*})]\right]\right. \\
&\qquad\quad + (1-\tilde{Y}_i)\left[(1-Z_{i^*})\,\mathbb{E}_{\boldsymbol{\beta},\boldsymbol{\omega},\boldsymbol{Se},\boldsymbol{Sp}}(\log Sp_{i^*}) + Z_{i^*}\,\mathbb{E}_{\boldsymbol{\beta},\boldsymbol{\omega},\boldsymbol{Se},\boldsymbol{Sp}}[\log(1-Sp_{i^*})]\right] \\
&\qquad\quad \left.\left. + \tilde{Y}_i\,\mathbb{E}_{\boldsymbol{\beta},\boldsymbol{\omega}}(\eta_i)\right\}\right\}.
\end{aligned}
$$

Under the Master Pool Testing (MPT) protocol with a pool size of 3. For MPT, we make the same relaxing assumption as for DT. We also assume $\boldsymbol{Se}$ and $\boldsymbol{Sp}$ are known.

For $Z_j = 0$, $q^{(t)}(\tilde{Y}_l, \tilde{Y}_m, \tilde{Y}_n)$ is same as under the DT, except that any expectations involing $\boldsymbol{Se}$ or $\boldsymbol{Sp}$ are replaced with the known constants $\boldsymbol{Se}$ and $\boldsymbol{Sp}$. Thus

$$q^{(t)}\left(\tilde{Y}_l, \tilde{Y}_m, \tilde{Y}_n\right) \propto \exp\left\{\mathbb{I}\left(\sum_{i\in\mathcal{P}_j} \tilde{Y}_i > 0\right)\log\left(1 - Se_j\right)\right.$$
$$\left. + \left[1 - \mathbb{I}\left(\sum_{i\in\mathcal{P}_j} \tilde{Y}_i > 0\right)\right](\log Sp_j) + \sum_{i\in\mathcal{P}_j} \tilde{Y}_i\, \mathbb{E}_{\boldsymbol{\beta},\boldsymbol{\omega}}(\eta_i)\right\}.$$

Since no retesting is performed under MPT, when the corresponding pool response $Z_j = 1$, we have:

$$q^{(t)}\left(\tilde{Y}_l, \tilde{Y}_m, \tilde{Y}_n\right) \propto \exp\left\{\mathbb{I}\left(\sum_{i\in\mathcal{P}_j} \tilde{Y}_i > 0\right)\log Se_j\right.$$
$$+ \left[1 - \mathbb{I}\left(\sum_{i\in\mathcal{P}_j} \tilde{Y}_i > 0\right)\right]\log(1 - Sp_j)$$
$$\left. + \tilde{Y}_i\, \mathbb{E}_{\boldsymbol{\beta},\boldsymbol{\omega}}(\eta_i)\right\}\Bigg\}.$$

Under Individual Testing (IT). We again assume that $\boldsymbol{Se}, \boldsymbol{Sp}$ are known.

When the individual pool response $Z_i = 0$,

$$q^{(t)}\left(\tilde{Y}_i\right) \propto \exp\left\{\tilde{Y}_i \log\left(1 - Se_{(i)}\right)\right.$$
$$\left. + \left(1 - \tilde{Y}_i\right)\log Sp_{(i)} + \tilde{Y}_i \mathbb{E}_{\boldsymbol{\beta},\boldsymbol{\omega}}\left(\eta_i\right)\right\}.$$

When the individual pool response $Z_i = 1$,

$$q^{(t)}\left(\tilde{Y}_i\right) \propto \exp\left\{\tilde{Y}_i \log Se_{(i)}\right.$$
$$\left. + \left(1 - \tilde{Y}_i\right)\log\left(1 - Sp_{(i)}\right) + \tilde{Y}_i \mathbb{E}_{\boldsymbol{\beta},\boldsymbol{\omega}}\left(\eta_i\right)\right\}.$$

Appendix A.2. Derivation of Expectations of Approximation Densities

Since the closed form for the optimal variational factor $q^t(\tilde{\boldsymbol{Y}})$ is available under $Z_j = 0$ or $Z_j = 1$, and $\mathbb{E}_{\boldsymbol{\beta},\boldsymbol{\omega}}\left(\eta_i\right) = \boldsymbol{x}_i^\top \mathbb{E}_\beta(\boldsymbol{\beta})$, (see Appendix A.1), the expectation of $\tilde{\boldsymbol{Y}}$ can be derived as follows.

For $i \in \mathcal{P}_j$ and $\mathcal{P}_j = \{l, m, n\}$, we can calculate $\mathbb{E}(\tilde{Y}_i)$ by marginalizing over $q^{(t)}\left(\tilde{Y}_l, \tilde{Y}_m, \tilde{Y}_n\right)$:

$$\mathbb{E}(\tilde{Y}_i) = \frac{p^*_{\tilde{Y}_i=1,0,0} + p^*_{\tilde{Y}_i=1,1,0} + p^*_{\tilde{Y}_i=1,0,1} + p^*_{\tilde{Y}_i=1,1,1}}{\sum_{x=0}^{1}\sum_{y=0}^{1}\sum_{z=0}^{1} p^*_{xyz}}.$$

where

$$p^*_{\tilde{y}_l,\tilde{y}_m,\tilde{y}_n} = \frac{p_{\tilde{y}_l,\tilde{y}_m,\tilde{y}_n}}{\prod_{i\in\mathcal{P}_j}\left[1 - g^{-1}(\eta_i)\right]}.$$

After this, we can calculate $\mathbb{E}(\tilde{Z}_j)$, that is

$$\mathbb{E}(\tilde{Z}_j) = 1 - \frac{p^*_{0,0,0}}{\sum_{x=0}^{1}\sum_{y=0}^{1}\sum_{z=0}^{1} p^*_{xyz}}$$

Recall that $q^{(t)}(\boldsymbol{\beta}) \sim \mathcal{N}_p\left(\boldsymbol{\mu}^*_b, \Sigma^*_b\right)$ and thus $\mathbb{E}(\boldsymbol{\beta}) = \boldsymbol{\mu}^*_b$.

For each i, we have $q^{(t)}(\omega_i) \sim \mathrm{PG}(1, \xi_i)$ and thus $\mathbb{E}(\omega_i) = \frac{1}{2\xi_i}\tanh(\xi_i/2)$.

For $m = 1, .., M$, we have that $q^{(t)}(Se_m) \sim \text{Beta}\left(a^*_{e(m)}, b^*_{e(m)}\right)$ and $q^{(t)}(Sp_m) \sim \text{Beta}\left(a^*_{p(m)}, b^*_{p(m)}\right)$. Appealing to the well-known formula for the expectation of the natural log of a beta random variable, we have that

$$\begin{aligned}
\mathbb{E}\left(\log Se_{(m)}\right) &= \psi\left(a^{*(t)}_{e(m)}\right) - \psi\left(a^{*(t)}_{e(m)} + b^{*(t)}_{e(m)}\right),\\
\mathbb{E}\left[\log(1 - Se_{(m)})\right] &= \psi\left(b^{*(t)}_{e(m)}\right) - \psi\left(a^{*(t)}_{e(m)} + b^{*(t)}_{e(m)}\right),\\
\mathbb{E}\left(\log Sp_{(m)}\right) &= \psi\left(a^{*(t)}_{p(m)}\right) - \psi\left(a^{*(t)}_{p(m)} + b^{*(t)}_{p(m)}\right),\\
\mathbb{E}\left[\log(1 - Sp_{(m)})\right] &= \psi\left(b^{*(t)}_{p(m)}\right) - \psi\left(a^{*(t)}_{p(m)} + b^{*(t)}_{p(m)}\right).
\end{aligned}$$

where $\psi(\cdot)$ is the digamma function.

Appendix B. Derivation of the Covariance Matrix for the Hybrid Inference Approach

By the missing information principle and Louis's method, the observed Fisher information matrix can be expressed as

$$\begin{aligned}
\mathcal{I}(\boldsymbol{\theta}) =& -\mathbb{E}\left\{\frac{\partial^2}{\partial\boldsymbol{\theta}\partial\boldsymbol{\theta}^\top}\mathcal{L}(\boldsymbol{\theta} \mid \mathbf{Z}) \mid \mathbf{Z}\right\}\\
&= -\mathbb{E}\left\{\frac{\partial^2}{\partial\boldsymbol{\theta}\partial\boldsymbol{\theta}^\top}\mathcal{L}(\boldsymbol{\theta} \mid \tilde{\boldsymbol{Y}}, \mathbf{Z}) \mid \mathbf{Z}\right\} - \mathrm{cov}\left\{\frac{\partial}{\partial\boldsymbol{\theta}}\mathcal{L}(\boldsymbol{\theta} \mid \tilde{\boldsymbol{Y}}, \mathbf{Z}) \mid \mathbf{Z}\right\}.
\end{aligned}$$

Appendix B.0.1. 1. Known Sensitivity *and* Specificity

Under known sensitivity and specificity, we define the parameter $\boldsymbol{\theta} = \boldsymbol{\beta}$ and the log-likelihood function is

$$
\begin{aligned}
\mathcal{L}(\boldsymbol{\beta} \mid \tilde{\boldsymbol{Y}}, \mathbf{Z}) =& \sum_{j=1}^{J} \left\{ \tilde{Z}_j C_{1j} + (1 - \tilde{Z}_j) C_{2j} \right\} \\
&+ \sum_{i=1}^{N} \left\{ \tilde{Y}_i \log \left[g^{-1}(\eta_i) \right] + (1 - \tilde{Y}_i) \log \left[1 - g^{-1}(\eta_i) \right] \right\}, \\
g^{-1}(\eta_i) =& p_i = \frac{\exp\left(\boldsymbol{x}_i^\top \boldsymbol{\beta} \right)}{1 + \exp\left(\boldsymbol{x}_i^\top \boldsymbol{\beta} \right)}, \\
1 - g^{-1}(\eta_i) =& q_i = \frac{1}{1 + \exp\left(\boldsymbol{x}_i^\top \boldsymbol{\beta} \right)},
\end{aligned}
$$

where $C_{1j} = Z_j \log Se_j + (1 - Z_j) \log(1 - Se_j)$ and $C_{2j} = Z_j \log(1 - Sp_j) + (1 - Z_j) \log(Sp_j)$.

For the first-order partial derivatives with respect to the parameter $\boldsymbol{\beta}$,

$$
\begin{aligned}
\frac{\partial}{\partial \boldsymbol{\beta}} \mathcal{L}(\boldsymbol{\beta} \mid \tilde{\boldsymbol{Y}}, \mathbf{Z}) &= \frac{\partial}{\partial \boldsymbol{\beta}} \left(\sum_{i=1}^{N} \tilde{Y}_i \log p_i + (1 - \tilde{Y}_i) \log q_i \right) \\
&= \sum_{i=1}^{N} \boldsymbol{x}_i^\top \left(\tilde{Y}_i - p_i \right).
\end{aligned}
$$

For the second-order partial derivatives with respect to the parameter $\boldsymbol{\beta}$,

$$
\frac{\partial^2}{\partial \boldsymbol{\beta} \partial \boldsymbol{\beta}^\top} \mathcal{L}(\boldsymbol{\beta} \mid \tilde{\boldsymbol{Y}}, \mathbf{Z}) = - \sum_{i=1}^{N} p_i q_i \boldsymbol{x}_i^\top \boldsymbol{x}_i.
$$

Therefore, for the expectations of the second-order partial derivatives, we have

$$
\mathbb{E} \left(\frac{\partial^2 \mathcal{L}}{\partial \boldsymbol{\beta} \partial \boldsymbol{\beta}^\top} \right) = - \sum_{i=1}^{N} \hat{p}_i \hat{q}_i \boldsymbol{x}_i^\top \boldsymbol{x}_i,
$$

where

$$
\hat{p}_i = \frac{\exp\left[\boldsymbol{x}_i^\top \mathbb{E}(\boldsymbol{\beta}) \right]}{1 + \exp\left[\boldsymbol{x}_i^\top \mathbb{E}(\boldsymbol{\beta}) \right]} \text{ and } \hat{q}_i = \frac{1}{1 + \exp\left[\boldsymbol{x}_i^\top \mathbb{E}(\boldsymbol{\beta}) \right]}.
$$

For the covariance matrix of the first-order derivatives, we have

$$\mathrm{Cov}\left(\frac{\partial \mathcal{L}}{\partial \boldsymbol{\beta}}\right) = \mathbf{X}^\top \,\mathrm{Cov}(\tilde{\mathbf{Y}})\mathbf{X}.$$

Here, $\mathbf{X}$ is the $N \times p$ design matrix and the covariance matrix of $\tilde{\mathbf{Y}}$, denoted as $\mathrm{Cov}(\tilde{\mathbf{Y}}) = \boldsymbol{\Sigma}$, can be expressed in block form to account for within-pool and cross-pool dependencies:

$$\boldsymbol{\Sigma}_{il} = \begin{cases} \mathbb{E}(\tilde{Y}_i)\left[1 - \mathbb{E}(\tilde{Y}_i)\right], & \text{if } i = l, \\ \mathbb{E}(\tilde{Y}_i\tilde{Y}_l) - \mathbb{E}(\tilde{Y}_i)\mathbb{E}(\tilde{Y}_l), & \text{if } i \neq l, \text{ and } i, l \text{ in the same pool}, \\ 0, & \text{if } i \neq l, \text{ and } i, l \text{ in different pools}, \end{cases}$$

where

$$\mathbb{E}(\tilde{Y}_i\tilde{Y}_l) = \Pr\left(\tilde{Y}_{i=1}, \tilde{Y}_{l=1} \mid \boldsymbol{Z}\right) = \frac{p_{i=1,l=1,1} + p_{i=1,l=1,0}}{\sum_{i=0}^{l}\sum_{l=0}^{1}\sum_{m=0}^{1} p_{ilm}},$$

and p_{ilm} is the probability are defined in Appendix A.1.

Appendix B.1. 2. Unknown Sensitivity and Specificity

Under unknown sensitivity and specificity, we have parameter vector $\boldsymbol{\theta} = (\boldsymbol{\beta}, \boldsymbol{Se}, \boldsymbol{Sp})^\top$ and the log-likelihood function is

$$\begin{aligned} \mathcal{L}(\boldsymbol{\beta}, \boldsymbol{Se}, \boldsymbol{Sp} \mid \mathbf{Z}, \tilde{\boldsymbol{Y}}) = \sum_{j=1}^{J} \Big\{ & \tilde{Z}_j \left[Z_j \log Se_j + (1 - Z_j)\log(1 - Se_j)\right] \\ & + (1 - \tilde{Z}_j)\left[Z_j \log(1 - Sp_j) + (1 - Z_j)\log Sp_j\right] \Big\} \\ & + \sum_{i=1}^{N}\left[\tilde{Y}_i \log p_i + (1 - \tilde{Y}_i)\log q_i\right]. \end{aligned}$$

Assume we have $\mathcal{M} = 2$ assays, i.e., one assay for pool testing and other other for individual retesting, then we have $Se_j = Se_{(p)}$ for all pooled testing $j = 1, \ldots, J^*$, and $Se_j = Se_{(i)}$, for all retesting pools $j = J^* + 1, ..., J$. We define a similar framework for the Sp_j. Thus, the log-likelihood function incorporating $Se_{(p)}, Se_{(i)}, Sp_{(p)}, Sp_{(i)}$ and retesting information can be updated

as

$$
\begin{aligned}
\mathcal{L}(\boldsymbol{\beta}, \boldsymbol{Se}, \boldsymbol{Sp} \mid \mathbf{Z}, \tilde{\boldsymbol{Y}}) = & \sum_{j=1}^{J} \Big\{ \tilde{Z}_j \left[Z_j \log Se_{(p)} + (1 - Z_j) \log(1 - Se_{(p)}) \right] \\
& + (1 - \tilde{Z}_j) \left[Z_j \log(1 - Sp_{(p)}) + (1 - Z_j) \log Sp_{(p)} \right] \Big\} \\
& + \sum_{i \in \mathcal{P}_{J+}} \Big\{ \tilde{Y}_i \left[Y_i \log Se_{(i)} + (1 - Y_i) \log(1 - Se_{(i)}) \right] \\
& + (1 - \tilde{Y}_i) \left[Y_i \log(1 - Sp_{(i)}) + (1 - Y_i) \log Sp_{(i)} \right] \Big\} \\
& + \sum_{i=1}^{N} \left[\tilde{Y}_i \log p_i + (1 - \tilde{Y}_i) \log q_i \right].
\end{aligned}
$$

The first-order and second-order derivatives with respect to $\boldsymbol{\beta}$ are the same as those derived before. In this section, we focus on the first-order and second-order derivatives with respect to $Se_{(p)}, Se_{(i)}, Sp_{(p)}$ and $Sp_{(i)}$. For the first-order derivatives, we have

$$
\begin{aligned}
\frac{\partial \mathcal{L}}{\partial Se_{(p)}} &= \frac{1}{Se_{(p)}(1 - Se_{(p)})} \sum_{j=1}^{J} \tilde{Z}_j (Z_j - Se_{(p)}), \\
\frac{\partial \mathcal{L}}{\partial Se_{(i)}} &= \frac{1}{Se_{(i)}(1 - Se_{(i)})} \sum_{i \in \mathcal{P}_{J+}} \tilde{Y}_i (Y_i - Se_{(i)}), \\
\frac{\partial \mathcal{L}}{\partial Sp_{(p)}} &= \frac{1}{Sp_{(p)}(1 - Sp_{(p)})} \sum_{j=1}^{J} (1 - \tilde{Z}_j)(1 - Z_j - Sp_{(p)}), \\
\frac{\partial \mathcal{L}}{\partial Sp_{(i)}} &= \frac{1}{Sp_{(i)}(1 - Sp_{(i)})} \sum_{i \in \mathcal{P}_{J+}} (1 - \tilde{Y}_i)(1 - Y_i - Sp_{(i)}).
\end{aligned}
$$

For the second-order derivatives, we have

$$
\begin{aligned}
\frac{\partial^2 \mathcal{L}}{\partial Se_{(p)}^2} &= \frac{1}{\left[Se_{(p)}(1-Se_{(p)}\right]^2} \sum_{j=1}^{J} -\tilde{Z}_j \left(Se_{(p)}^2 + Z_j - 2Z_j Se_{(p)}\right), \\
\frac{\partial^2 \mathcal{L}}{\partial Se_{(i)}^2} &= \frac{1}{\left[Se_{(i)}(1-Se_{(i)}\right]^2} \sum_{i\in\mathcal{P}_{J+}} -\tilde{Y}_i \left(Se_{(i)}^2 + Y_i - 2Y_i Se_{(i)}\right), \\
\frac{\partial^2 \mathcal{L}}{\partial Sp_{(p)}^2} &= \frac{1}{\left[Sp_{(p)}(1-Sp_{(p)}\right]^2} \sum_{j=1}^{J} -(1-\tilde{Z}_j) \left[Sp_{(p)}^2 + (1-Z_j) - 2(1-Z_j) Sp_{(p)}\right], \\
\frac{\partial^2 \mathcal{L}}{\partial Sp_{(i)}^2} &= \frac{1}{\left[Sp_{(i)}(1-Sp_{(i)}\right]^2} \sum_{i\in\mathcal{P}_{J+}} -(1-\tilde{Y}_i) \left[Sp_{(i)}^2 + (1-Y_i) - 2(1-Y_i) Sp_{(i)}\right].
\end{aligned}
$$

Therefore, we have the expectations of those derivatives as follows,

$$
\begin{aligned}
\mathbb{E}\left(\frac{\partial^2 \mathcal{L}}{\partial Se_{(p)}^2}\right) &= \frac{1}{\left[Se_{(p)}(1-Se_{(p)}\right]^2} \sum_{j=1}^{J} -\mathbb{E}(\tilde{Z}_j) \left(Se_{(p)}^2 + Z_j - 2Z_j Se_{(p)}\right), \\
\mathbb{E}\left(\frac{\partial^2 \mathcal{L}}{\partial Se_{i}^2}\right) &= \frac{1}{[Se_i(1-Se_i)]^2} \sum_{i\in\mathcal{P}_{J+}} -\mathbb{E}(\tilde{Y}_i) \left(Se_{(i)}^2 + Y_i - 2Y_i Se_{(i)}\right), \\
\mathbb{E}\left(\frac{\partial^2 \mathcal{L}}{\partial Sp_{(p)}^2}\right) &= \frac{1}{\left[Sp_{(p)}(1-Sp_{(p)}\right]^2} \sum_{j=1}^{J} -[1-\mathbb{E}(\tilde{Z}_j)] \left[Sp_{(p)}^2 + (1-Z_j) - 2(1-Z_j) Sp_{(p)}\right], \\
\mathbb{E}\left(\frac{\partial^2 \mathcal{L}}{\partial Sp_{(i)}^2}\right) &= \frac{1}{[Sp_{(i)}(1-Sp_{(i)})]^2} \sum_{i\in\mathcal{P}_{J+}} -[1-\mathbb{E}(\tilde{Y}_i)] \left(Sp_{(i)}^2 + (1-Y_i) - 2(1-Y_i) Sp_{(i)}\right).
\end{aligned}
$$

Additionally,

$$
\begin{aligned}
&\mathbb{E}\left(\frac{\partial^2 \mathcal{L}}{\partial Se_{(p)} \partial Sp_{(p)}}\right) = \mathbb{E}\left(\frac{\partial^2 \mathcal{L}}{\partial Se_{(p)} \partial Se_{(i)}}\right) = \mathbb{E}\left(\frac{\partial^2 \mathcal{L}}{\partial Se_{(p)} \partial Sp_{(i)}}\right) = 0, \\
&\mathbb{E}\left(\frac{\partial^2 \mathcal{L}}{\partial Sp_{(p)} \partial Se_{(i)}}\right) = \mathbb{E}\left(\frac{\partial^2 \mathcal{L}}{\partial Sp_{(p)} \partial Sp_{(i)}}\right) = \mathbb{E}\left(\frac{\partial^2 \mathcal{L}}{\partial Se_{(i)} \partial Sp_{(i)}}\right) = 0, \\
&\mathbb{E}\left(\frac{\partial^2 \mathcal{L}}{\partial Se_{(p)} \partial \boldsymbol{\beta}}\right) = \mathbb{E}\left(\frac{\partial^2 \mathcal{L}}{\partial Se_{(i)} \partial \boldsymbol{\beta}}\right) = \mathbb{E}\left(\frac{\partial^2 \mathcal{L}}{\partial Sp_{(p)} \partial \boldsymbol{\beta}}\right) = \mathbb{E}\left(\frac{\partial^2 \mathcal{L}}{\partial Sp_{(i)} \partial \boldsymbol{\beta}}\right) = 0.
\end{aligned}
$$

The covariance of the first-order derivatives with respect to $\boldsymbol{\beta}$ is the same as the previous section. We focus on the covariance of first derivatives with

respect to $Se_{(p)}, Se_{(i)}, Sp_{(p)}$ and $Sp_{(i)}$. We have

$$\mathrm{Var}\left(\frac{\partial \mathcal{L}}{\partial Se_{(p)}}\right) = \frac{1}{\left[Se_{(p)}(1-Se_{(p)})\right]^2} \sum_{j=1}^{J} (Z_j - Se_{(p)})^2 \mathbb{E}(\tilde{Z}_j)[1-\mathbb{E}(\tilde{Z}_j)],$$

$$\mathrm{Var}\left(\frac{\partial \mathcal{L}}{\partial Sp_{(p)}}\right) = \frac{1}{\left[Sp_{(p)}(1-Sp_{(p)})\right]^2} \sum_{j=1}^{J} (1 - Z_j - Sp_{(p)})^2 \mathbb{E}(\tilde{Z}_j)\left[1-\mathbb{E}(\tilde{Z}_j)\right],$$

$$\mathrm{Var}\left(\frac{\partial \mathcal{L}}{\partial Se_{(i)}}\right) = \frac{1}{\left[Se_{(i)}(1-Se_{(i)})\right]^2} \sum_{j=1}^{J^+} \mathrm{Var}\left[\sum_{i\in\mathcal{P}_j} \tilde{Y}_i (Y_i - Se_{(i)})\right],$$

$$\mathrm{Var}\left(\frac{\partial \mathcal{L}}{\partial Sp_{(i)}}\right) = \frac{1}{\left[Sp_{(i)}(1-Sp_{(i)})\right]^2} \sum_{j=1}^{J^+} \mathrm{Var}\left[\sum_{i\in\mathcal{P}_j} (1-\tilde{Y}_i)(1 - Y_i - Sp_{(i)})\right],$$

where

$$\begin{aligned}\mathrm{Var}\left[\sum_{i\in\mathcal{P}_j} \tilde{Y}_i (Y_i - Se_{(i)})\right] &= \sum_{i\in\mathcal{P}_j} (Y_i - Se_{(i)})^2 \mathbb{E}(\tilde{Y}_i)[1-\mathbb{E}(\tilde{Y}_i)] \\ &+ \sum_{l\neq i, k\in\mathcal{P}_j} (Y_i - Se_{(i)})(Y_l - Se_{(i)}) \mathbf{\Sigma}_{il}, \\ \mathrm{Var}\left[\sum_{i\in\mathcal{P}_j} (1-\tilde{Y}_i)(1 - Y_i - Sp_{(i)})\right] &= \sum_{i\in\mathcal{P}_j} (1 - Y_i - Sp_{(i)})^2 \mathbb{E}(\tilde{Y}_i)[1-\mathbb{E}(\tilde{Y}_i)] \\ &+ \sum_{l\neq i, l\in\mathcal{P}_j} (1 - Y_i - Sp_{(i)})(1 - Y_l - Sp_{(i)}) \mathbf{\Sigma}_{il}.\end{aligned}$$

For the covariance terms, we have

$$
\begin{aligned}
\mathrm{Cov}\left(\frac{\partial \mathcal{L}}{\partial Se_{(p)}}, \frac{\partial \mathcal{L}}{\partial Sp_{(p)}}\right) =& \frac{1}{Se_{(p)}(1-Se_{(p)})}\frac{1}{Sp_{(p)}(1-Sp_{(p)})} \\
&\times \sum_{j=1}^{J} -(Z_j - Se_{(p)})(1 - Z_j - Sp_{(p)})\mathbb{E}(\tilde{Z}_j)\left[1-\mathbb{E}(\tilde{Z}_j)\right], \\
\mathrm{Cov}\left(\frac{\partial \mathcal{L}}{\partial Se_{(p)}}, \frac{\partial \mathcal{L}}{\partial Se_{(i)}}\right) =& \frac{1}{Se_{(p)}(1-Se_{(p)})}\frac{1}{Se_{(i)}(1-Se_{(i)})} \\
&\times \sum_{j}^{J^+}\sum_{l\in\mathcal{P}_j}(Z_j - Se_{(p)})(Y_l - Se_{(i)})\mathbb{E}(\tilde{Y}_l)\left[1-\mathbb{E}(\tilde{Z}_j)\right], \\
\mathrm{Cov}\left(\frac{\partial \mathcal{L}}{\partial Se_{(p)}}, \frac{\partial \mathcal{L}}{\partial Sp_{(i)}}\right) =& \frac{1}{Se_{(p)}(1-Se_{(p)})}\frac{1}{Sp_{(i)}(1-Sp_{(i)})} \\
&\times \sum_{j}^{J^+}\sum_{l\in\mathcal{P}_j}-(Z_j - Se_{(p)})(1 - Y_l - Sp_{(i)})\mathbb{E}(\tilde{Y}_l)\left[1-\mathbb{E}(\tilde{Z}_j)\right], \\
\mathrm{Cov}\left(\frac{\partial \mathcal{L}}{\partial Sp_{(p)}}, \frac{\partial \mathcal{L}}{\partial Se_{(i)}}\right) =& \frac{1}{Sp_{(p)}(1-Sp_{(p)})}\frac{1}{Se_{(i)}(1-Se_{(i)})} \\
&\times \sum_{j}^{J^+}\sum_{l\in\mathcal{P}_j}-(1 - Z_j - Se_{(p)})(Y_l - Se_{(i)})\mathbb{E}(\tilde{Y}_l)\left[1-\mathbb{E}(\tilde{Z}_j)\right], \\
\mathrm{Cov}\left(\frac{\partial \mathcal{L}}{\partial Sp_{(p)}}, \frac{\partial \mathcal{L}}{\partial Sp_{(i)}}\right) =& \frac{1}{Sp_{(p)}(1-Sp_{(p)})}\frac{1}{Sp_{(i)}(1-Sp_{(i)})} \\
&\times \sum_{j}^{J^+}\sum_{l\in\mathcal{P}_j}(1 - Z_j - Sp_{(p)})(1 - Y_l - Sp_{(i)})\mathbb{E}(\tilde{Y}_l)\left[1-\mathbb{E}(\tilde{Z}_j)\right], \\
\mathrm{Cov}\left(\frac{\partial \mathcal{L}}{\partial Se_{(i)}}, \frac{\partial \mathcal{L}}{\partial Sp_{(i)}}\right) =& \frac{1}{Se_{(i)}(1-Se_{(i)})}\frac{1}{Sp_{(i)}(1-Sp_{(i)})} \\
&\times \sum_{j\in\mathcal{P}_{J+}}\sum_{l\in\mathcal{P}_j}(Y_j - Se_{(i)})(1 - Y_l - Sp_{(i)})\mathbf{\Sigma}_{jl}.
\end{aligned}
$$

Regarding the covariance terms between the first-order derivatives respect to $Se_{(p)}, Se_{(i)}, Sp_{(p)}, Sp_{(i)}$ and the first-order derivatives with respect to $\boldsymbol{\beta}$, we

have

$$\text{Cov}\left(\frac{\partial\mathcal{L}}{\partial Se_{(p)}}, \frac{\partial\mathcal{L}}{\partial\boldsymbol{\beta}}\right) = \frac{1}{Se_{(p)}(1-Se_{(p)})}\sum_{j=1}^{J}(Z_j - Se_{(p)})\sum_{i\in\mathcal{P}_j}\boldsymbol{x}_i^\top\left[1-\mathbb{E}(\tilde{Z}_j)\right]\mathbb{E}(\tilde{Y}_i),$$

$$\text{Cov}\left(\frac{\partial\mathcal{L}}{\partial Sp_{(p)}}, \frac{\partial\mathcal{L}}{\partial\boldsymbol{\beta}}\right) = \frac{1}{Sp_{(p)}(1-Sp_{(p)})}\sum_{j=1}^{J}-(1-Z_j - Sp_{(p)})\sum_{i\in\mathcal{P}_j}\boldsymbol{x}_i^\top\left[1-\mathbb{E}(\tilde{Z}_j)\right]\mathbb{E}(\tilde{Y}_i),$$

$$\text{Cov}\left(\frac{\partial\mathcal{L}}{\partial Se_{(i)}}, \frac{\partial\mathcal{L}}{\partial\boldsymbol{\beta}}\right) = \frac{1}{Se_{(i)}(1-Se_{(i)})}\sum_{j\in\mathcal{P}_{J+}}\sum_{l\in\mathcal{P}_j}(Y_j - Se_{(i)})\boldsymbol{x}_j^\top\boldsymbol{\Sigma}_{jl},$$

$$\text{Cov}\left(\frac{\partial\mathcal{L}}{\partial Sp_{(i)}}, \frac{\partial\mathcal{L}}{\partial\boldsymbol{\beta}}\right) = \frac{1}{Sp_{(i)}(1-Sp_{(i)})}\sum_{j\in\mathcal{P}_{J+}}\sum_{l\in\mathcal{P}_j}-(1-Y_j - Sp_{(i)})\boldsymbol{x}_j^\top\boldsymbol{\Sigma}_{jl}.$$

Appendix C. Additional Simulation Study Information

Appendix C.1. Algorithm Initializations

Both the VB and the MCMC algorithms require parameter initializations. For the MPT and IT with known $\boldsymbol{Se}$ and $\boldsymbol{Sp}$ scenarios, the VB algorithm is initialized by taking $\mathbb{E}(\tilde{Y}_i)^{(0)} = 0$ and $\mathbb{E}(\omega_i)^{(0)} = \frac{1}{4}$ for all i; $\mathbb{E}(\boldsymbol{\beta})^{(1)}$ is then computed based on these values. For the DT scenarios with unknown $\boldsymbol{Se}$ and $\boldsymbol{Sp}$, the $\mathbb{E}(\tilde{Y}_i)^{(0)}s$, $\mathbb{E}(\omega_i)^{(0)}s$ and $\mathbb{E}(\boldsymbol{\beta})^{(1)}$ are computed in the same manner as for MPT and IT. However, for the DT scenarios computing the $\mathbb{E}(\tilde{Y}_i)^{(1)}$s requires not only $\mathbb{E}(\boldsymbol{\beta})^{(0)}$, but also $\mathbb{E}(\log(\boldsymbol{Se}))^{(0)}$, $\mathbb{E}(\log(1-\boldsymbol{Se}))^{(0)}$, $\mathbb{E}(\log(\boldsymbol{Sp}))^{(0)}$ and $\mathbb{E}(\log(1-\boldsymbol{Sp}))^{(0)}$. These parameters are randomly initialized by sampling $a_{e(1)}$, $b_{e(1)}$, $a_{e(2)}$, $b_{e(2)}$, $a_{p(1)}$, $b_{p(1)}$, $a_{p(2)}$, and $b_{p(2)}$ from normal distributions and applying formulas for $\mathbb{E}(\log(\boldsymbol{Se}))^{(0)}$, $\mathbb{E}(\log(1-\boldsymbol{Se}))^{(0)}$, $\mathbb{E}(\log(\boldsymbol{Sp}))^{(0)}$ and $\mathbb{E}(\log(1-\boldsymbol{Sp}))^{(0)}$ provided in Appendix A.2.

For the MPT and IT scenarios, the MCMC algorithm is initialized by taking $\boldsymbol{\beta}^{(0)} = (0,0,0)^\top$ and randomly initializing the $\tilde{Y}_i^{(0)}$s from a Bernoulli(p_i) distribution, where $p_i = \exp(\boldsymbol{x}_i\boldsymbol{\beta}^{(0)})/(1+\exp(\boldsymbol{x}_i\boldsymbol{\beta}^{(0)})$. For the DT scenarios, $\boldsymbol{\beta}^{(0)}$ and the $\tilde{Y}_i^{(0)}$s are initialized as in the MPT and IT scenarios and $\boldsymbol{Se}$ and $\boldsymbol{Sp}$ are initialized by sampling $a_{e(1)}$, $b_{e(1)}$, $a_{e(2)}$, $b_{e(2)}$, $a_{p(1)}$, $b_{p(1)}$, $a_{p(2)}$, and $b_{p(2)}$ from normal distributions and setting $Se_{(m)}^{(0)} = a_{e(m)}/(a_{e(m)} + b_{e(m)})$ and $Sp_{(m)}^{(0)} = a_{p(m)}/(a_{p(m)} + b_{p(m)})$ for $m = 1, 2$.

Appendix C.2. Additional Simulation Study Results

Figures C.1 - C.6 contain additional simulation study results to allow for a careful comparison of the runtime for the two algorithms. For each of the scenarios described in the main manuscript, 100 datasets were generated. The VB algorithm was applied to each dataset using increasingly strict stopping tolerance thresholds $\epsilon = 10^0, 10^{-1}, 10^{-2}, \ldots, 10^{-8}$ with a maximum of 50000 allowed VB iterations. The VB algorithm terminated due to the change in the ELBO being less than ϵ rather than due to reaching the maximum number of allowed iterations for all datasets.

The MCMC algorithm was run using burn-in periods of 1000, 2000,...,10000. A posterior sample of size 5000 was obtained after each burn-in period, so that the total number of MCMC iterations ranged from 6000 to 15000. We initially considered additional MCMC configurations with shorter overall run time (e.g. smaller posterior sample sizes and/or shorter burn-in periods), but found that these faster configurations performed poorly in many scenarios, having a high empirical bias, low empirical coverage probabilities and small effective sample sizes.

Both algorithms were run on the University of South Carolina's Hyperion high performance computing cluster using an Intel(R) Xeon(R) Platinum 8358 CPU @ 2.60GHz with 64 cores and 256GB of RAM. To ensure a consistent computational environment and minimize thread contention, both algorithms were restricted to one thread per dataset.

For each configuration of each algorithm, we compute the average absolute bias and empirical coverage probability for all parameters. The hybrid inference approach is used to produce Wald confidence intervals for the VB algorithm. To succinctly summarize performance, we then take the maximum of the average absolute bias over the three regression coefficients, i.e., $\boldsymbol{\beta}$, and the minimum of the empirical coverage probabilities over the regression coefficients from each configuration and plot these values versus the configuration's average runtime. Figures C.1 and C.2 contain the absolute bias results from the MPT and DT scenarios, respectively. Figures C.3 and C.4 contain the empirical coverage probability results for MPT and DT, respectively. Figures C.5 and C.6 contain the maximum absolute bias and minimum empirical coverage probabilities of any of the four assay accuracy parameters, i.e., $\boldsymbol{Se}$ and $\boldsymbol{Sp}$, from the DT scenarios. In the figures, triangular plotting symbols are used for the VB algorithm while circles are used for the MCMC algorithm. Color is used to distinguish between different configurations within each algorithm. For the MCMC algorithm, color denotes the

Table D.1: Demographics characteristics of All of US research program participants included in the data application.

	Total n(%) or Mean (SD)	COVID-19 Positive n(%) or Mean (SD)	p-value
N	43183 (100)	3585 (8.30)	-
Age	53.85 (16.34)	51.56 (16.23)	< 0.001
Race/Ethnicity			
Black	7656 (17.73)	650 (8.49)	< 0.001
Hispanic	8045 (18.63)	952 (11.83)	
Other Races/ Unknown/Declined	3547 (8.21)	253 (7.13)	
White	23938 (55.43)	1730 (7.23)	
Education			
College One to Three	12269 (28.41)	1101 (8.97)	< 0.001
High School or GED	8834 (20.46)	859 (9.72)	
Less Than High School	3997 (9.26)	455 (11.38)	
Unknown/Declined	1329 (3.08)	109 (8.20)	
College and Above	16757 (38.80)	1061 (6.33)	
General Health			
Good or Fair	24850 (57.54)	2075 (8.35)	0.01
Poor	3100 (7.18)	207 (6.68)	
Unknown/Declined	709 (1.64)	63 (8.89)	
Excellent or Very Good	14527 (33.64)	1240 (8.57)	

length of the burn-in period, whereas for the VB algorithm, color denotes the stopping tolerance threshold.

Appendix D. Additional Results of Data Application

Table D.1 summarizes frequency counts for the demographic characteristics of the study population from the National Institutes of Health's All of Us Research Program. The p-values in this table are calculated using two-sample t-tests for continuous variables and chi-square tests for categorical variables.

Tables D.2 and D.3 present data application results for the VB method and MCMC algorithm under the MPT and IT protocols with known testing accuracy.

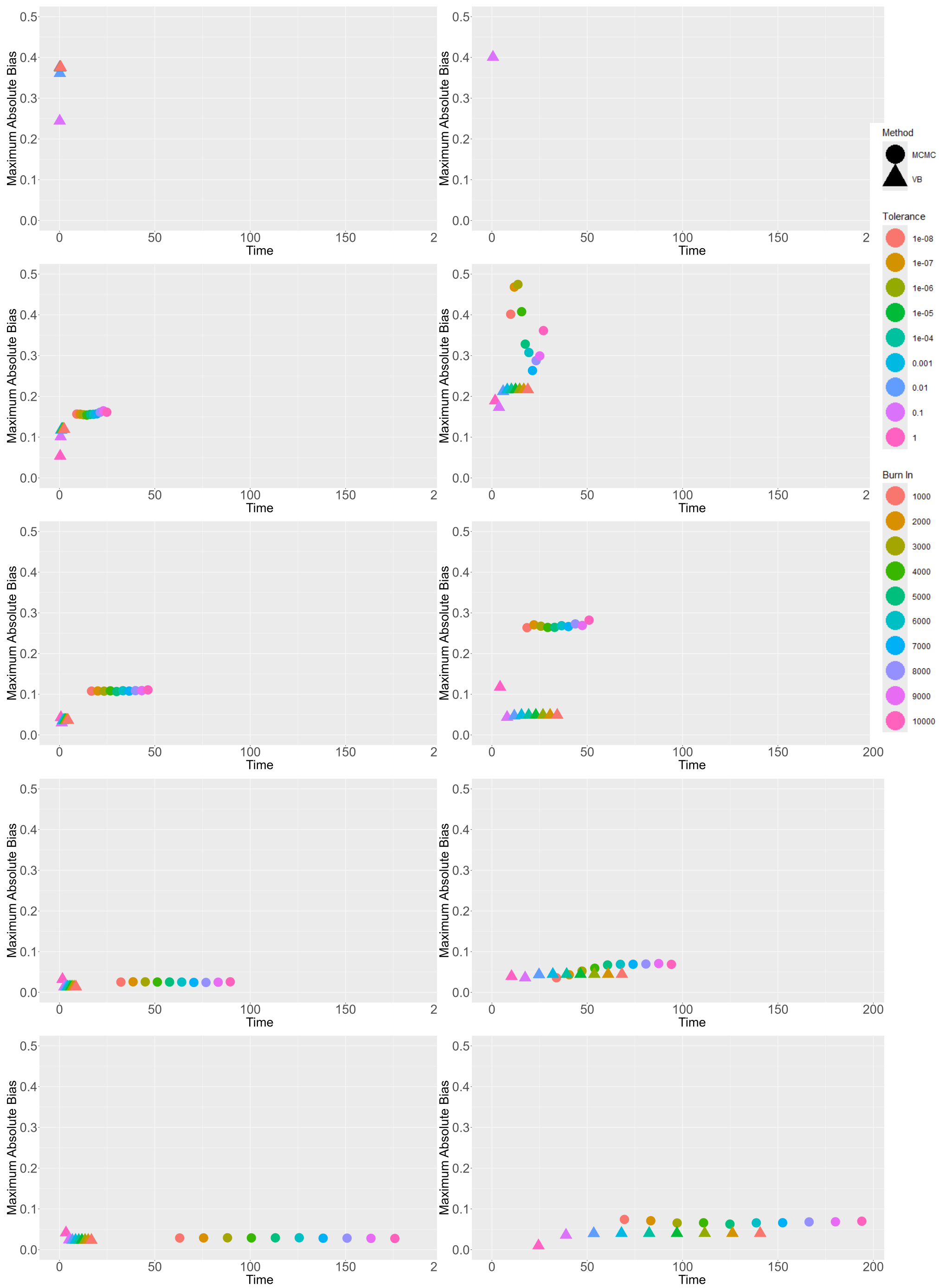

Figure C.1: The figure displays the maximum empirical absolute bias of any of the three regression coefficients under each configuration of the VB and MCMC algorithms for the MPT scenarios. From top to bottom, the rows correspond to sample sizes of $N = 300, 1500, 3000, 6000, 12000$ while the columns correspond to pool sizes of 3 (left) and 10 (right). The x-axis corresponds to average runtime in seconds. If a configuration's maximum absolute bias exceeds 0.5, it is not shown on the figure.

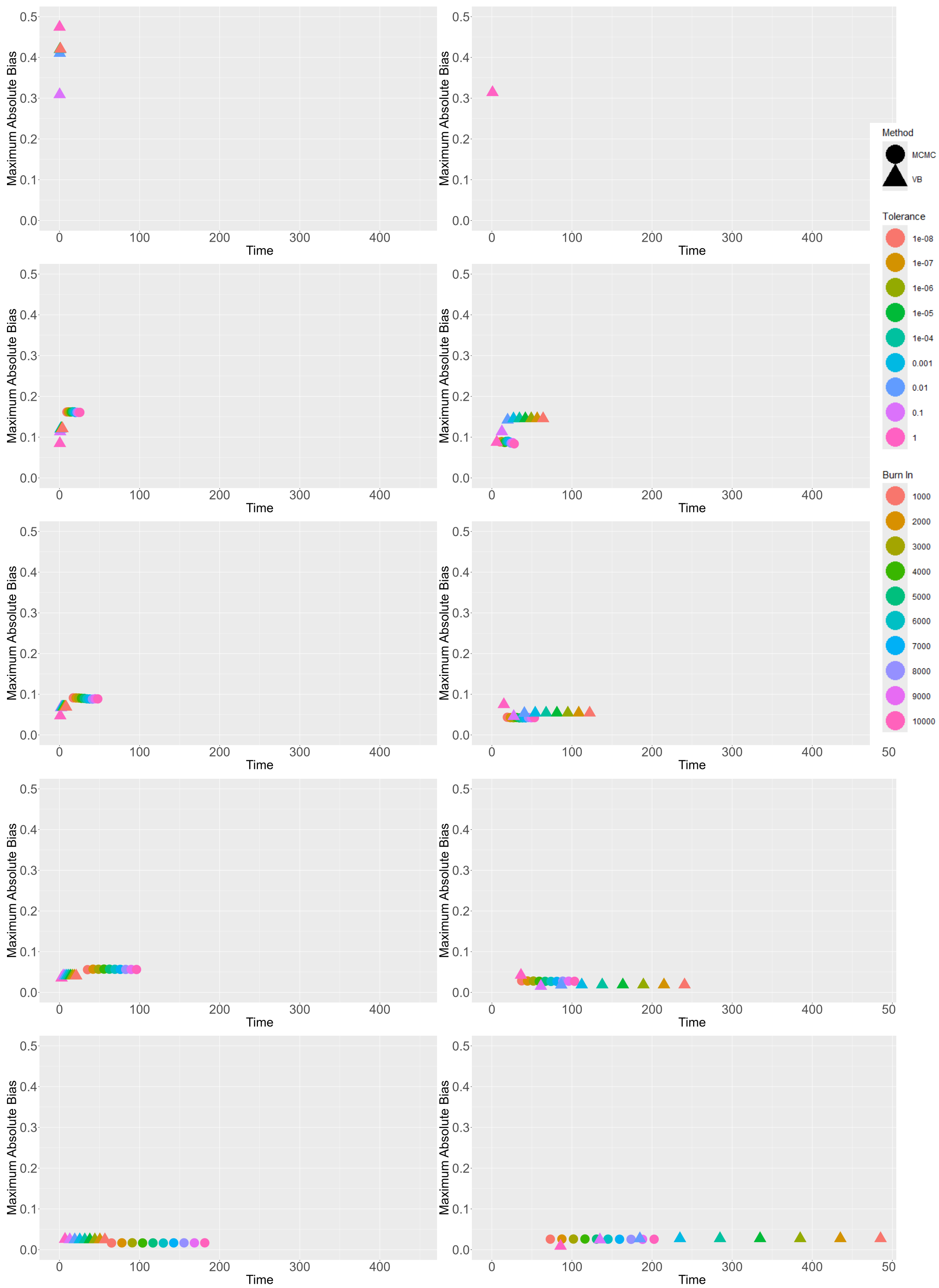

Figure C.2: The figure displays the maximum empirical absolute bias of any of the three regression coefficients under each configuration of the VB and MCMC algorithms for the DT scenarios. From top to bottom, the rows correspond to sample sizes of $N = 300, 1500, 3000, 6000, 12000$ while the columns correspond to pool sizes of 3 (left) and 10 (right). The x-axis corresponds to average runtime in seconds. If a configuration's maximum absolute bias exceeds 0.5, it is not shown on the figure.

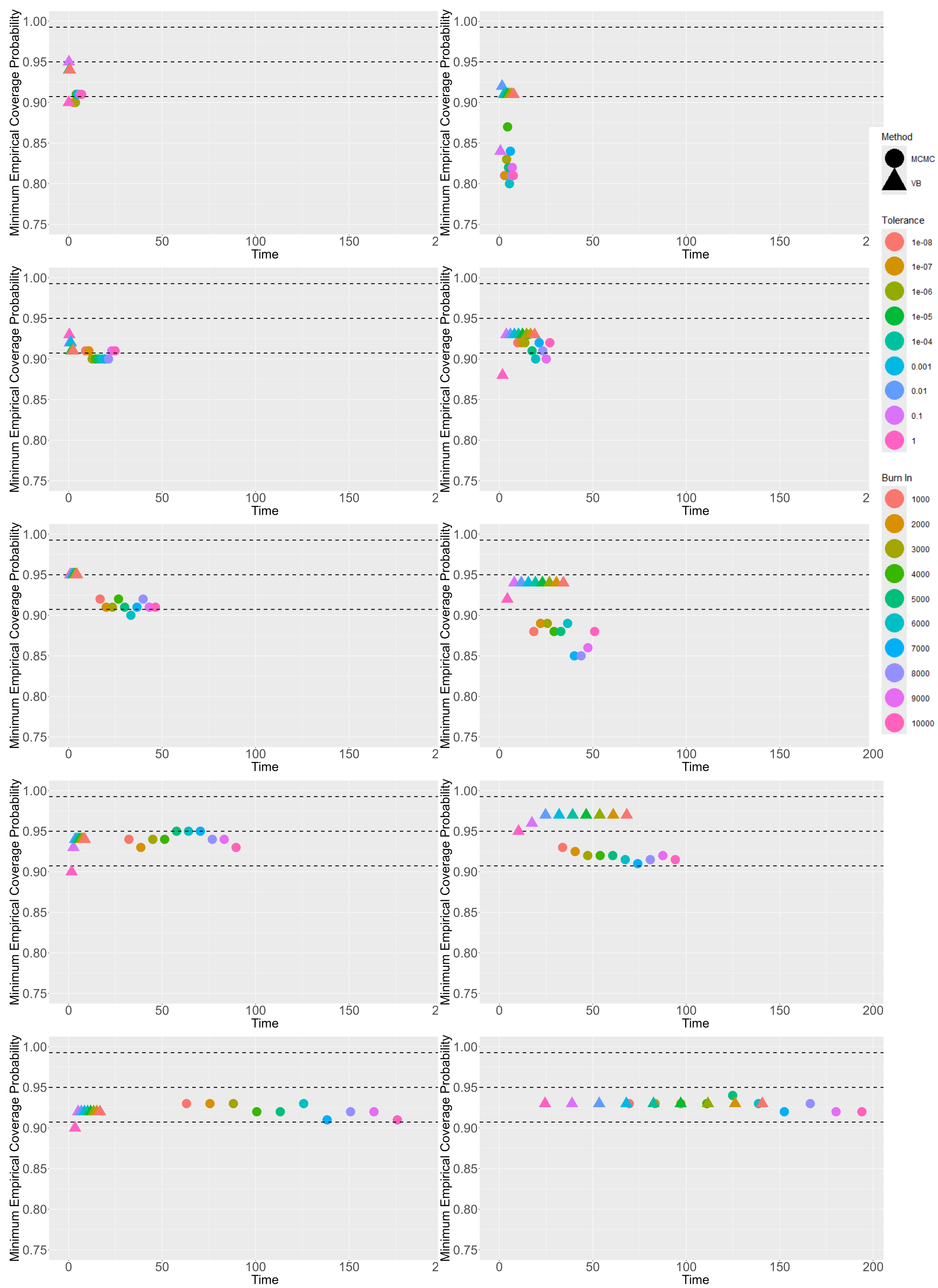

Figure C.3: The figure displays the minimum empirical coverage probability of any of the three regression coefficients under each configuration of the VB and MCMC algorithms for the MPT scenarios. From top to bottom, the rows correspond to sample sizes of $N = 300, 1500, 3000, 6000, 12000$ while the columns correspond to pool sizes of 3 (left) and 10 (right). The x-axis corresponds to average runtime in seconds. The dashed lines indicate the approximate limits of Monte Carlo error under the assumption that the true coverage probability is 0.95.

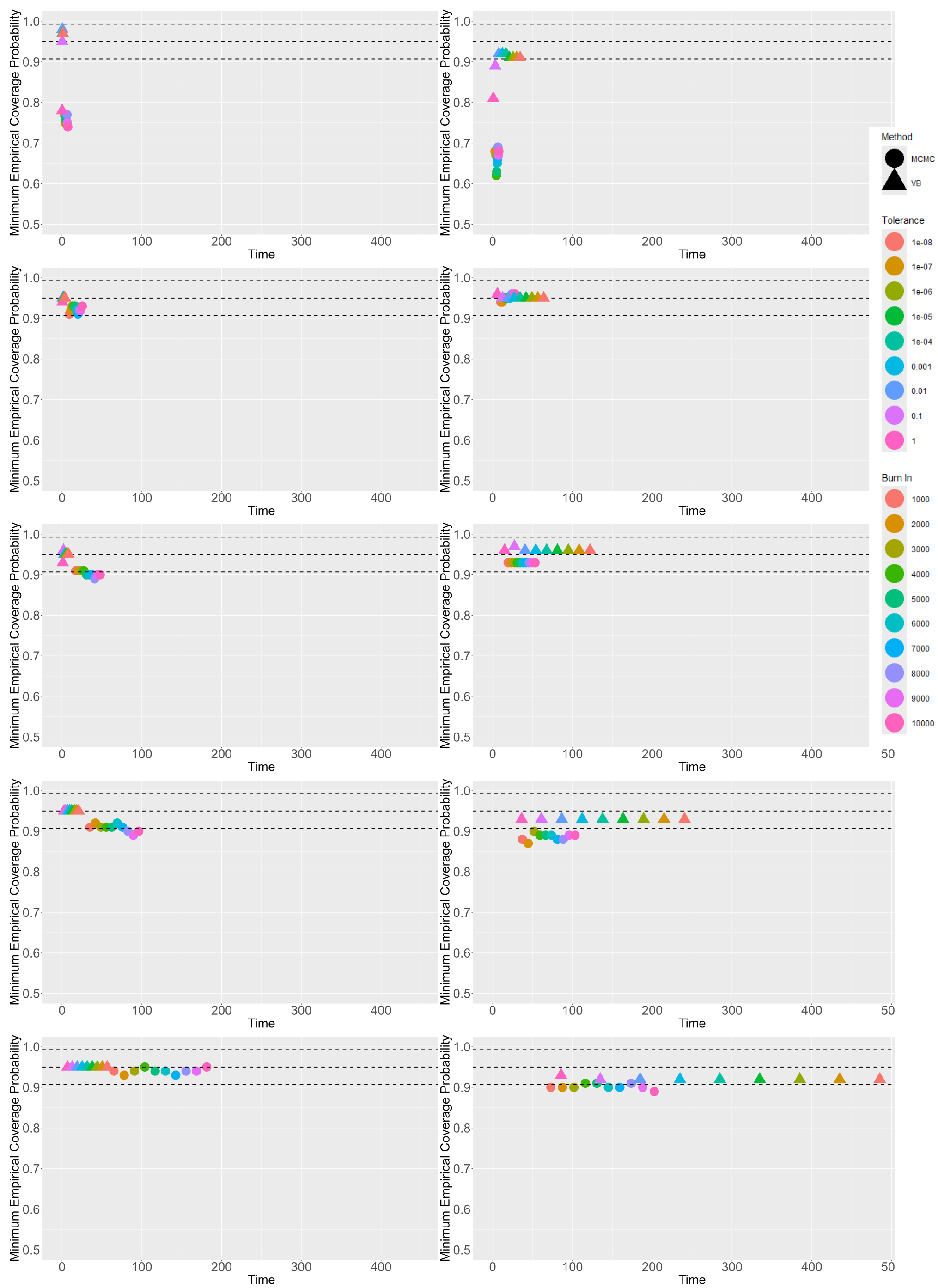

Figure C.4: The figure displays the minimum empirical coverage probability of any of the three regression coefficients under each configuration of the VB and MCMC algorithms for the DT scenarios. From top to bottom, the rows correspond to sample sizes of $N = 300, 1500, 3000, 6000, 12000$ while the columns correspond to pool sizes of 3 (left) and 10 (right). The x-axis corresponds to average runtime in seconds. The dashed lines indicate the approximate limits of Monte Carlo error under the assumption that the true coverage probability is 0.95.

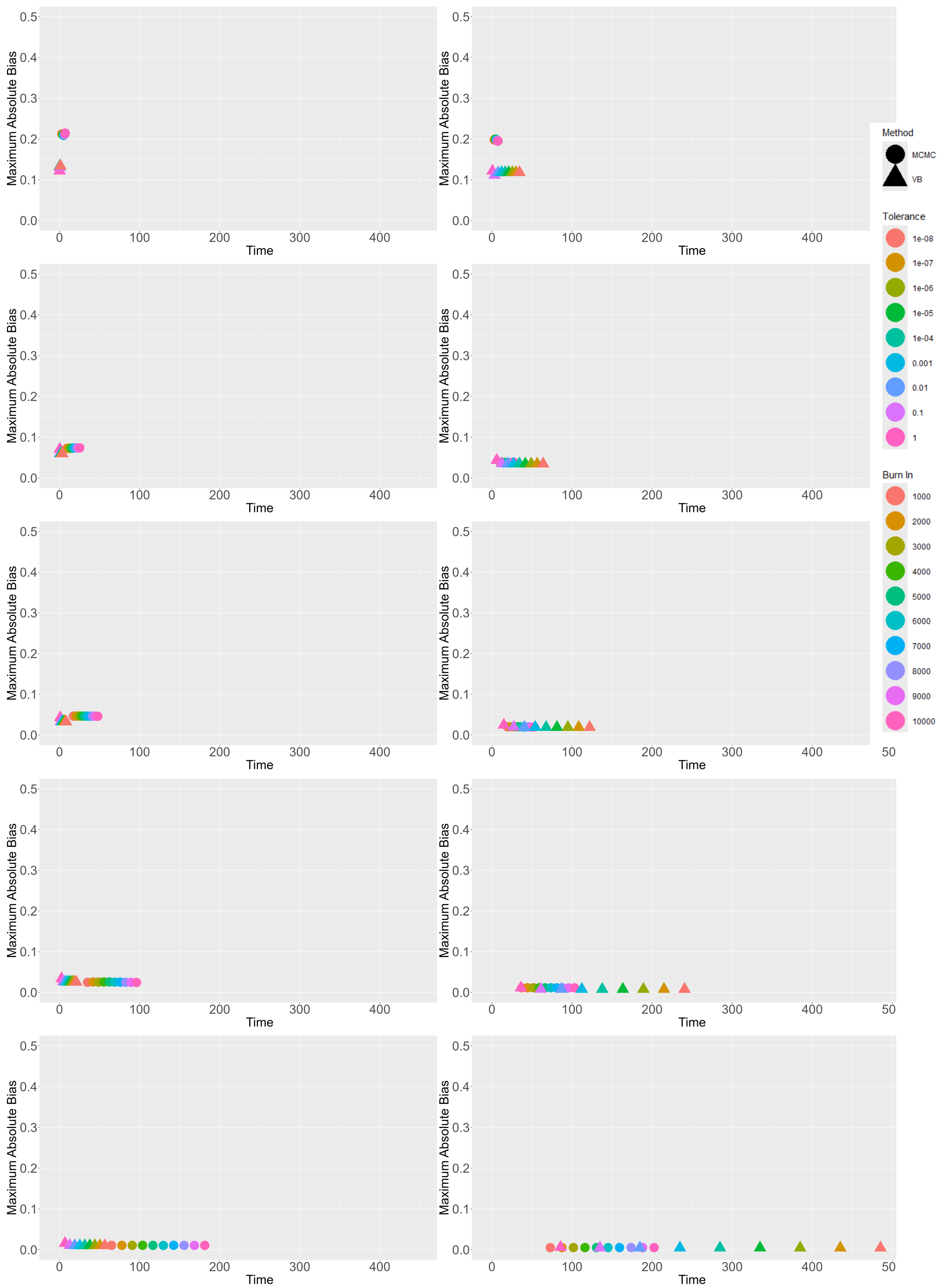

Figure C.5: The figure displays the maximum empirical absolute bias of any of the four assay accuracy parameters under each configuration of the VB and MCMC algorithms for the DT scenarios. From top to bottom, the rows correspond to sample sizes of $N = 300, 1500, 3000, 6000, 12000$ while the columns correspond to pool sizes of 3 (left) and 10 (right). The x-axis corresponds to average runtime in seconds. If a configuration's maximum absolute bias exceeds 0.5, it is not shown on the figure.

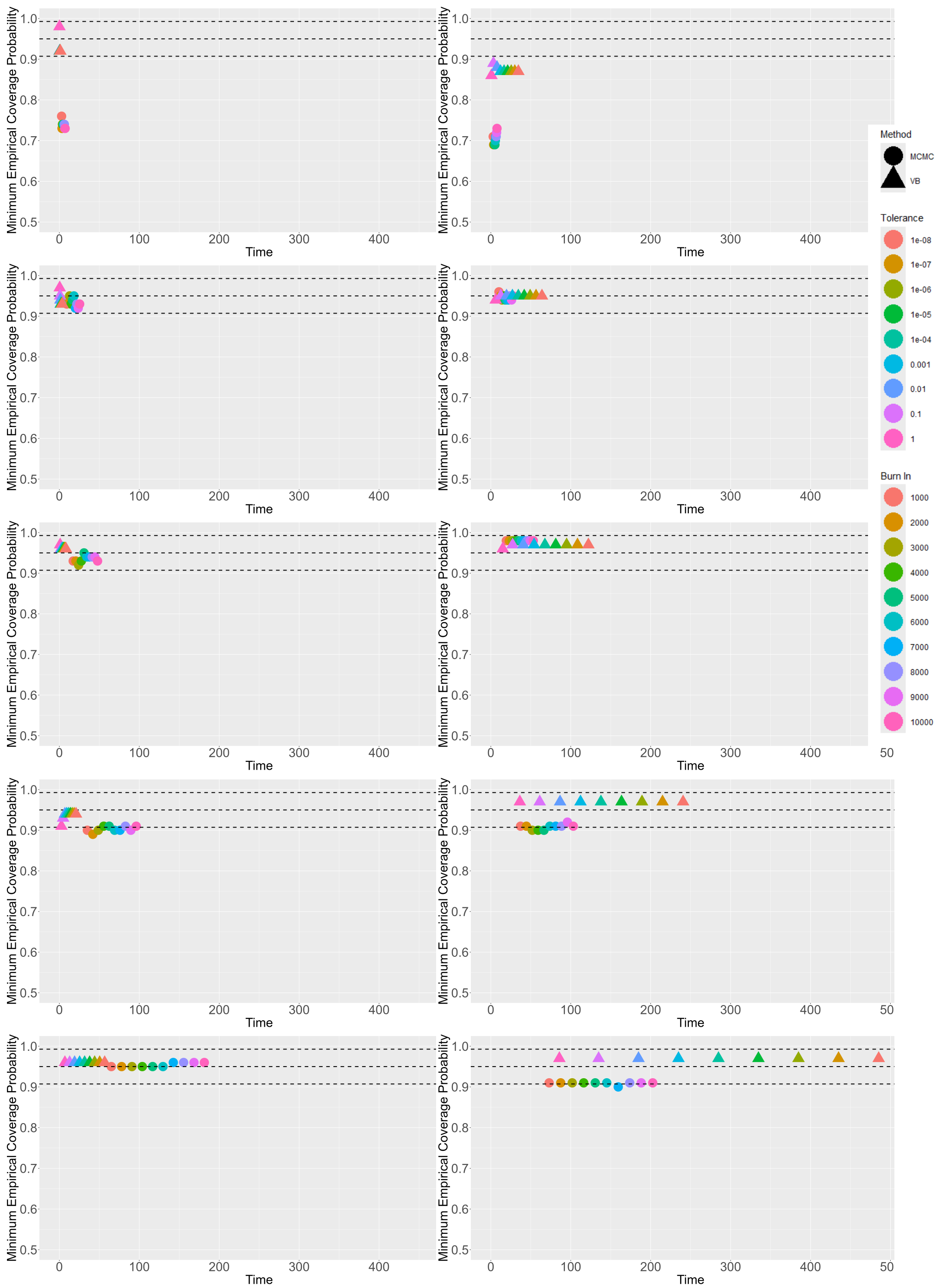

Figure C.6: The figure displays the minimum empirical coverage probability of any of the four assay accuracy parameters under each configuration of the VB and MCMC algorithms for the DT scenarios. From top to bottom, the rows correspond to sample sizes of $N = 300, 1500, 3000, 6000, 12000$ while the columns correspond to pool sizes of 3 (left) and 10 (right). The x-axis corresponds to average runtime in seconds. The dashed lines indicate the approximate limits of Monte Carlo error under the assumption that the true coverage probability is 0.95.

Table D.2: Results of variational Bayesian (VB) and Markov chain Monte Carlo (MCMC) method under the master pool testing (MPT) protocol with known sensitivity and specificity for 2019–2020 All of Us Research Program COVID data.

	**VB Method**					**MCMC Algorithm**		
Parameter	Estimate	SD	95% CI	HSD	95% HCI	Estimate	SD	95% CI
(Intercept)	-2.595	0.024	(-2.642 , -2.548)	0.065	(-2.722 , -2.469)	-2.608	0.064	(-2.729 , -2.481)
Age	-0.117	0.012	(-0.140 , -0.094)	0.031	(-0.178 , -0.056)	-0.116	0.029	(-0.173 , -0.059)
Race/Ethnicity								
Black	0.003	0.033	(-0.062 , 0.067)	0.093	(-0.180 , 0.185)	-0.003	0.091	(-0.188 , 0.174)
Hispanic	0.411	0.033	(0.346 , 0.475)	0.079	(0.255 , 0.566)	0.404	0.077	(0.251 , 0.557)
Other Races/ Unknown/Declined	-0.076	0.045	(-0.165 , 0.013)	0.133	(-0.338 , 0.186)	-0.072	0.132	(-0.338 , 0.171)
White	ref	ref	ref	ref	ref	ref	ref	ref
Education								
College One to Three	0.313	0.030	(0.255 , 0.371)	0.084	(0.149 , 0.477)	0.333	0.080	(0.168 , 0.487)
High School or GED	0.293	0.033	(0.227 , 0.358)	0.093	(0.110 , 0.475)	0.304	0.097	(0.102 , 0.482)
Less Than High School	0.412	0.045	(0.323 , 0.500)	0.111	(0.193 , 0.630)	0.423	0.110	(0.195 , 0.634)
Unknown/Declined	0.639	0.068	(0.505 , 0.773)	0.159	(0.328 , 0.950)	0.641	0.158	(0.323 , 0.938)
College and Above	ref	ref	ref	ref	ref	ref	ref	ref
General Health								
Good or Fair	-0.085	0.026	(-0.135 , -0.035)	0.069	(-0.220 , 0.050)	-0.087	0.071	(-0.217 , 0.061)
Poor	-0.614	0.050	(-0.712 , -0.515)	0.167	(-0.942 , -0.286)	-0.620	0.166	(-0.968 , -0.317)
Unknown/Declined	-0.295	0.095	(-0.480 , -0.109)	0.275	(-0.833 , 0.244)	-0.372	0.285	(-0.994 , 0.133)
Excellent or Very Good	ref	ref	ref	ref	ref	ref	ref	ref

Table D.3: Results of variational Bayesian (VB) and Markov chain Monte Carlo (MCMC) method under the individual testing (IT) protocol with known sensitivity and specificity for 2019–2020 All of Us Research Program COVID data.

	**VB Method**					**MCMC Algorithm**		
Parameter	Estimate	SD	95% CI	HSD	95% HCI	Estimate	SD	95% CI
(Intercept)	-2.604	0.024	(-2.651 , -2.557)	0.038	(-2.679 , -2.529)	-2.609	0.038	(-2.685 , -2.535)
Age	-0.105	0.012	(-0.128 , -0.082)	0.018	(-0.140 , -0.070)	-0.104	0.018	(-0.139 , -0.069)
Race/Ethnicity								
Black	0.045	0.033	(-0.020 , 0.109)	0.050	(-0.054 , 0.143)	0.044	0.050	(-0.053 , 0.143)
Hispanic	0.360	0.033	(0.295 , 0.425)	0.048	(0.267 , 0.454)	0.362	0.048	(0.268 , 0.454)
Other Races/ Unknown/Declined	-0.060	0.045	(-0.149 , 0.029)	0.072	(-0.202 , 0.082)	-0.062	0.074	(-0.202 , 0.087)
White	ref	ref	ref	ref	ref	ref	ref	ref
Education								
College One to Three	0.360	0.030	(0.302 , 0.418)	0.046	(0.269 , 0.451)	0.362	0.046	(0.273 , 0.449)
High School or GED	0.403	0.033	(0.338 , 0.469)	0.051	(0.303 , 0.503)	0.407	0.051	(0.303 , 0.505)
Less Than High School	0.529	0.045	(0.441 , 0.617)	0.065	(0.401 , 0.657)	0.534	0.064	(0.410 , 0.661)
Unknown/Declined	0.290	0.071	(0.151 , 0.428)	0.109	(0.076 , 0.503)	0.292	0.107	(0.082 , 0.499)
College and Above	ref	ref	ref	ref	ref	ref	ref	ref
General Health								
Good or Fair	-0.153	0.026	(-0.204 , -0.103)	0.039	(-0.230 , -0.077)	-0.151	0.039	(-0.226 , -0.074)
Poor	-0.468	0.049	(-0.565 , -0.371)	0.080	(-0.624 , -0.312)	-0.473	0.080	(-0.638 , -0.313)
Unknown/Declined	-0.042	0.092	(-0.221 , 0.138)	0.137	(-0.310 , 0.227)	-0.047	0.138	(-0.322 , 0.214)
Excellent or Very Good	ref	ref	ref	ref	ref	ref	ref	ref
